# Supplementary material for: Novel PFAS in Alligator Blood Discovered with Non-Targeted Ion Mobility-Mass Spectrometry
Source: bioRxiv. 2025 Mar 25:2025.03.20.644452. Preprint. [Version 1] doi: 10.1101/2025.03.20.644452 (PMC11974715; doi:10.1101/2025.03.20.644452)
Supplement: Supplementary file 1 [file media-2.pdf]

Supplemental document for

Novel PFAS in Alligators Discovered with Non-Targeted Ion Mobility-Mass Spectrometry

Anna K. Boatman[1], Gregory P. Kudzin[1], Kylie D. Rock[2], Matthew P. Guillette[3], Frank Robb[4], Scott M. Belcher[3], and Erin S. Baker[1*]

[1]Department of Chemistry, University of North Carolina at Chapel Hill, Chapel Hill, North Carolina 27514, USA

[2]Department of Biological Sciences, Clemson University, Clemson, South Carolina 29634, USA

[3]Department of Biological Sciences, North Carolina State University, Raleigh, North Carolina 27695, USA

[4]Environmental Education, Awareness, Research, Support & Services, Titusville, FL 32780, USA

* Corresponding authors

Contents

Supplemental Identification Information 4
Figure S1. MS1-only data supporting novel triether compounds. 4
Figure S2. Supporting evidence for Compound 628. 5
Figure S3. Supporting evidence for Compound 592. 6
Figure S4. MS1-only data supporting novel PFAESAs. 7
Figure S5. Supporting evidence for PFHpESA. 9
Figure S6. Supporting evidence for PFHxESA. 10
Figure S7. MS1-only data supporting co-br-PFUdA. 12
Figure S8. Supporting evidence for co-br-PFUdA. 13
Figure S9. MS1-only data supporting polyfluoroether sulfonic acid (PolyFESA) isomers. 14
Figure S10. Supporting evidence for NB2 isomer A. 15
Figure S11. Supporting evidence for NB2 isomer B. 16
Figure S12. Supporting evidence for Unknown PolyFESA C. 17
Figure S13. Supporting evidence for PolyFESA D. 18
Figure S14. MS1-only data supporting fipronil sulfone. 19
Figure S15. Supporting evidence for fipronil sulfone. 20
Figure S16. MS1-only data supporting U-PFOS. 21
Figure S17. Supporting evidence for U-PFOS. 22
Figure S18. Supporting evidence for PFPeDA. 23
Figure S19. Supporting evidence for PFUdS. 24
Figure S20. MS1-only fragmentation evidence of undetected PFECAs. 25
Supplemental Relative Abundance Evaluation 26
Figure S21. Preliminary temporal and spatial trend analysis for the 5 novel PFAS. 30
Figure S22. Relative abundance trends for PFSAs (PFEtS through PFOS). 32
Figure S23. Relative abundance trends for PFSAs (PFNS through PFDoS). 33
Figure S24. Relative abundance trends for PFCAs (PFHxA through PFUdA). 35
Figure S25. Relative abundance trends for PFCAs (PFDoA through PFHxDA). 37
Figure S26. Relative abundance trends for FTSs and PFASAs. 39
Figure S27. Relative abundance trends for Nafion byproducts. 40
Figure S28. Relative abundance trends for PFPis and diPAPs. 42
Figure S29. Relative abundance trends for 8:2 FTUCA and Cl-PFOS. 43
Figure S30. Relative abundance trends for novel triethers. 44
Figure S31. Relative abundance trends for novel PFAESAs. 45
Figure S32. Relative abundance trends for PFUdA isomers. 46
Figure S33. Relative abundance trends for PolyFESA isomers. 47
Figure S34. Relative abundance trends for fipronil sulfone. 48
Figure S35. Relative abundance trends for U-PFOS. 49
Figure S36. Relative abundance trends for PFPeDA and PFUdS. 50
Figure S37. Peak area trends for the unidentified features of interest. 52
LC-IMS-CID-HRMS Data Collection 53
Table SM1. LC gradient settings 53

Table SM2. Electrospray ionization source settings....53
Table SM3. IMS-MS settings....54
Table SM4. Collision energy ramp settings....54
Table SM5. Mass Profiler settings....54
Table SM6. Fluoromatch-IM settings....54
Supplemental Tables....**Error! Bookmark not defined.**

Supplemental Identification Information

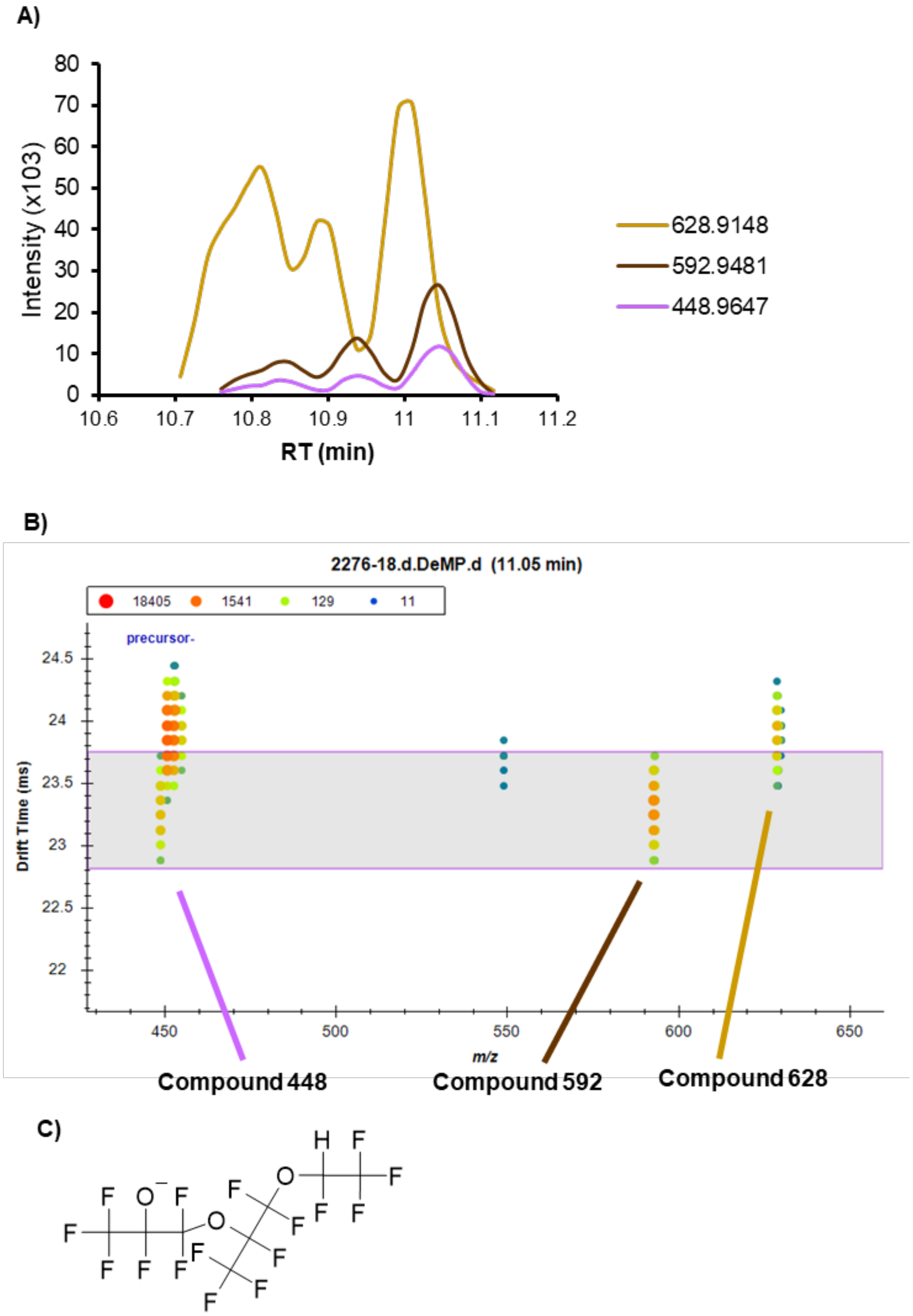

Figure S1. MS1-only data supporting novel triether compounds.

A) Extracted ion chromatogram (EIC) of a representative sample. **B)** Nested drift spectra for the predominant LC peak of all 3 compounds. **C)** Candidate structure for Compound 448. Together this supports Compound 448 as an in source fragment of Compound 592.

Identification and experimental measurements

- **Name:** Compound 628
- **PubChem CID:** n/a
- **Molecular formula:** C10H2F20O6S
- **Observed adduct(s):** [M-H]-
- **Exact *m/z*(s):** 628.9180
- **Measured CCS:** 176.00 Å^2
- **Measured RT:** 10.90 min (best peak, multiple peaks observed with lower RTs)

Supporting evidence

- **Mobility-aligned fragments:** 262.9760, 282.9822, 448.9676, 212.9792 (notably, no 281.9744 as seen in Cmpd 592)
- **Type of diagnostic evidence:** Transformation product of Compound 10 reported by Donelson *et al.*
- **Predicted CCS:** 176.51 Å^2 (reported by Donelson *et al.* for Compound 10 M-H-CO2)
- **Predicted RT:** > 7.9 min and multiple peaks (should be less polar than Compound 10)

Most likely candidate structure

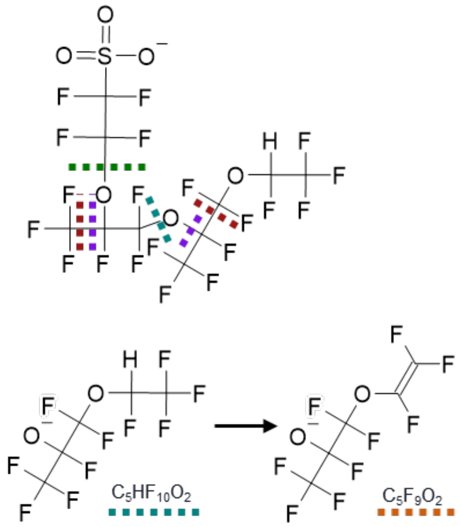

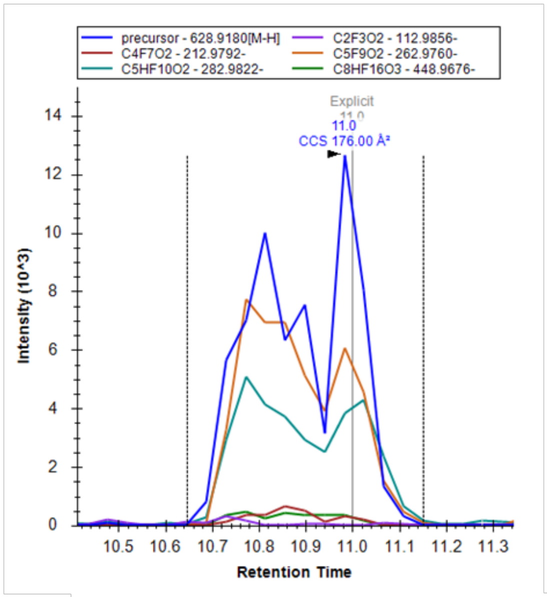

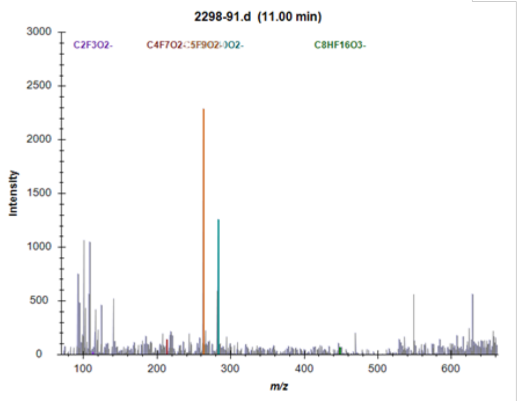

Level 3 checklist

☒ One or more structures
☒ Exact *m/z* and isotopic pattern match candidate structure
☒ CCS supported by library or predicted value
☒ RT supported by library or predicted value
☒ One or both support all candidates:
  - ☒ Mobility-aligned fragments
  - ☒ Diagnostic evidence

Figure S2. Supporting evidence for Compound 628.

Identification and experimental measurements

- **Name:** Compound 592
- **PubChem CID:** n/a
- **Molecular formula:** $C11H2F20O5$
- **Observed adduct(s):** [M-H]-
- **Exact *m/z*(s):** 592.9510
- **Measured CCS:** 173.20 Å^2
- **Measured RT:** 10.94 min (best peak, multiple peaks observed with lower RTs)

Supporting evidence

- **Mobility-aligned fragments:** 262.9760, 282.9822, 448.9676, 212.9792
- **Type of diagnostic evidence:** Transformation product of Compound 10 reported by Donelson *et al*.
- **Library / Predicted CCS:** select one and add
- **Library / Predicted RT:** select one and add

Most likely candidate structure

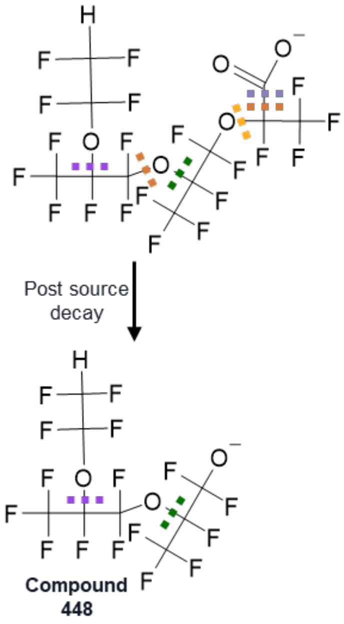

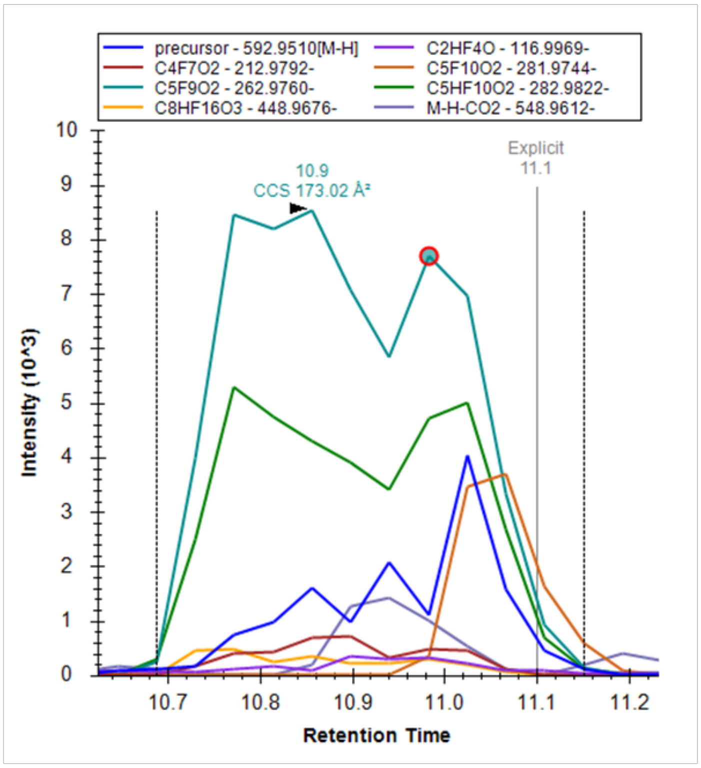

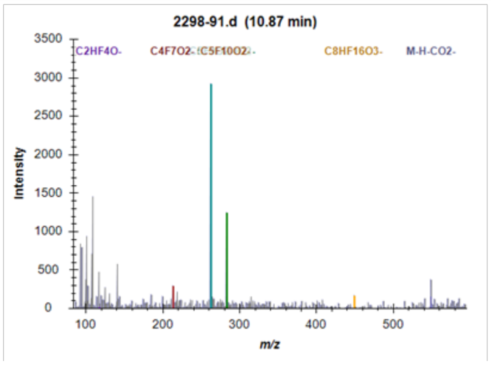

Level 3 checklist

☒ One or more structures
☒ Exact *m/z* and isotopic pattern match candidate structure
☒ CCS supported by library or predicted value
☒ RT supported by library or predicted value
☒ One or both support all candidates:
- ☒ Mobility-aligned fragments
- ☒ Diagnostic evidence

Figure S3. Supporting evidence for Compound 592.

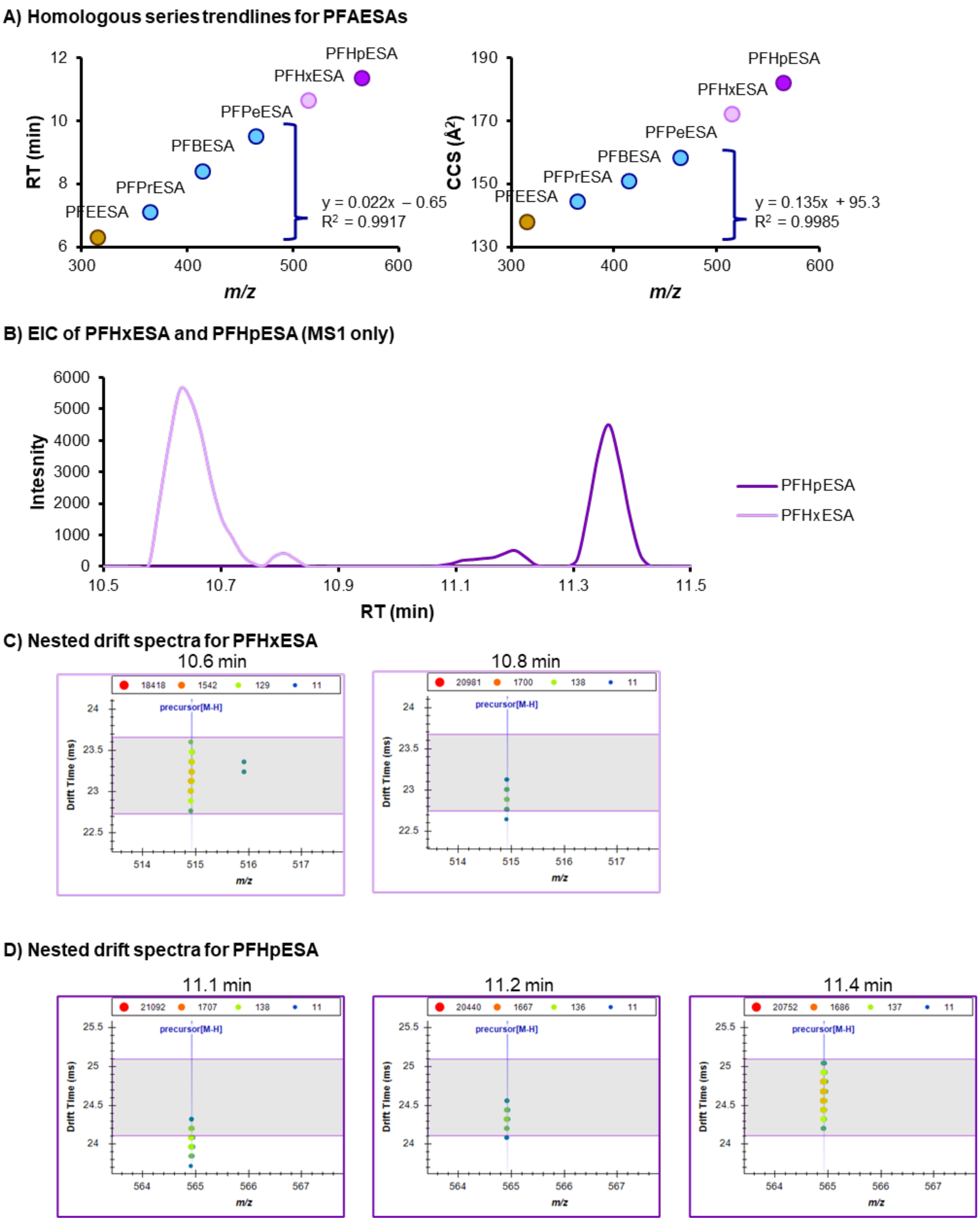

Figure S4. MS1-only data supporting novel PFAESAs.

A) RT and CCS vs *m/z* trendlines for the homologous series using library values for the smaller chain molecules. **B)** Extracted ion chromatogram (EIC) from a representative sample showing the multiple peaks detected. **C)** Nested drift spectra for PFHxESA. The lower CCS at the higher RT indicates that the ether linkage is different between the two

isomers. A smaller CCS indicates a more bent or compact structure while a higher RT is indicative of a longer fluoroalkyl tail, which would have more interactions with the C18 stationary phase compared to a tail with an ether linkage closer to the terminus. **D)** Nested drift spectra for PFHpESA.

Identification and experimental measurements
- **Name:** PFHpESA
- **PubChem CID:** multiple (see below)
- **Molecular formula:** $C_9HF_{19}O_4S$
- **Observed adduct(s):** [M-H]-
- **Exact *m/z*(s):** 564.9219
- **Measured CCS:** 181.89 Å^2
- **Measured RT:** 11.36 min (best peak)

Supporting evidence
- **Mobility-aligned fragments:** low intensity, not diagnostic
- **Type of diagnostic evidence:** Known exposure to homologous series; manufacturing knowledge
- **Predicted CCS:** 171.6 Å^2 (from homologous series trendline)
    - Difference (measured vs predicted): 5.7%
    - Supports different location of ether linkage
- **Predicted RT:** 11.8 min (from homologous series trendline)
    - Difference (measured vs predicted): -3.7%

Most likely candidate

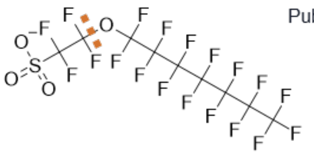

PubChem CID: n/a

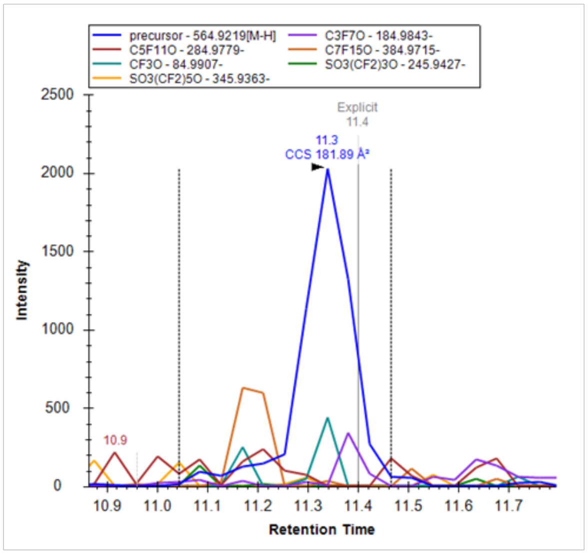

Other tentative candidates

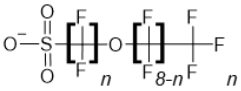

n = 1-8

PubChem CIDs:
- 139594233
- 139595773
- 139597196
- 168509881

Level 3 checklist
☒ One or more structures
☒ Exact *m/z* and isotopic pattern match candidate structure
☒ CCS supported by library or predicted value
☒ RT supported by library or predicted value
☒ One or both support all candidates:
 ☒ Mobility-aligned fragments
 ☒ Diagnostic evidence

Figure S5. Supporting evidence for PFHpESA.

Identification and experimental measurements

- **Name:** PFHxESA
- **PubChem CID:** multiple
- **Molecular formula:** $C_8HF_{17}O_4S$
- **Observed adduct(s):** [M-H]-
- **Exact *m/z*(s):** 514.9251
- **Measured CCS:** 172.00 Å^2
- **Measured RT:** 10.89 min (best peak)

Supporting evidence

- **Mobility-aligned fragments:** not diagnostic, low signal
- **Type of diagnostic evidence:** Known exposure to homologous series; manufacturing knowledge
- **Predicted CCS:** 164.8 Å^2 (homologous series trendline)
    - Difference (measured vs predicted): 4.2%
- **Predicted RT:** 10.7 min (homologous series trendline)
    - Difference (measured vs predicted): -0.4%

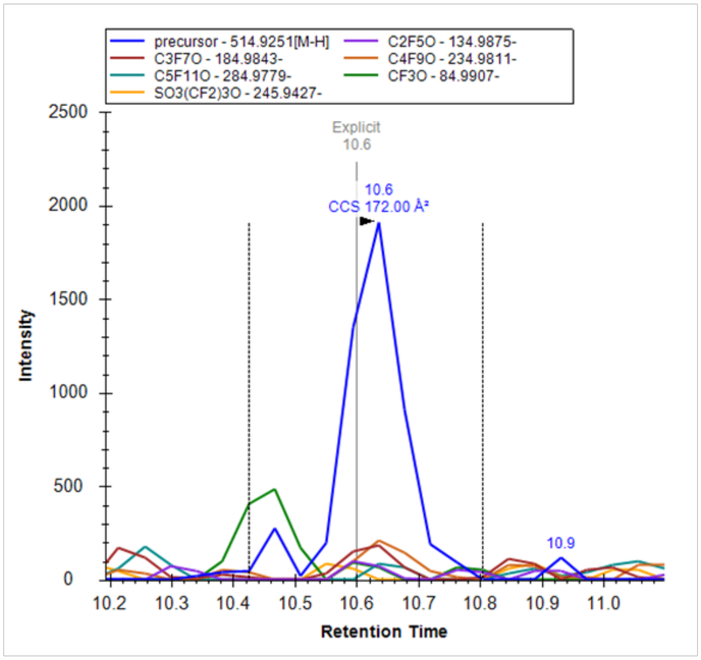

Most likely candidate

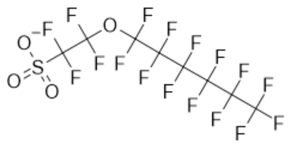

PubChem CID: 22568764

Other tentative candidates

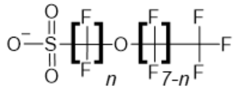

n = 1-7

PubChem CIDs:
- 139594536
- 139598276
- 88532751

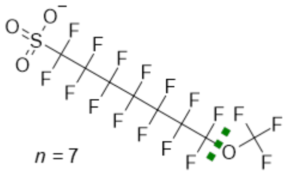

n = 7

PubChem CID: 139596291

Level 3 checklist

☒ One or more structures
☒ Exact *m/z* and isotopic pattern match candidate structure
☒ CCS supported by library or predicted value
☒ RT supported by library or predicted value
☒ One or both support all candidates:
    ☒ Mobility-aligned fragments
    ☒ Diagnostic evidence

Figure S6. Supporting evidence for PFHxESA.

A) EIC of all PFUdA isomers in a representative sample

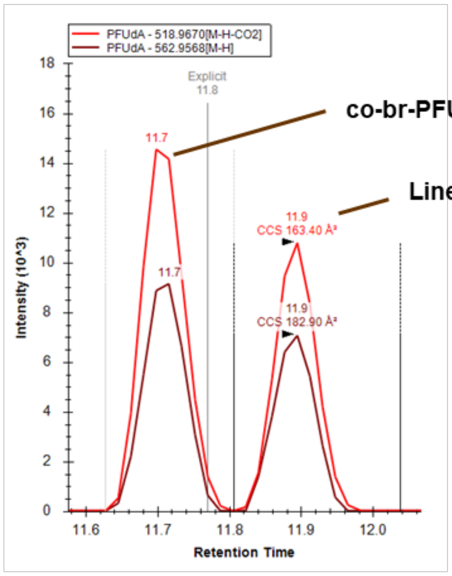

co-br-PFUdA (Level 3)

Linear PFUdA (Level 1, confirmed by reference standard)

A higher abundance of the $[M\text{-}H\text{-}CO_2]^-$ adduct is typical of all PFCAs with fewer than 12 carbons on our platform

B) Nested drift spectra of M-H- with drift window drawn using the CCS for l-PFUdA

The center of the peak is slightly below that of the linear, which is typical of branched PFCAs

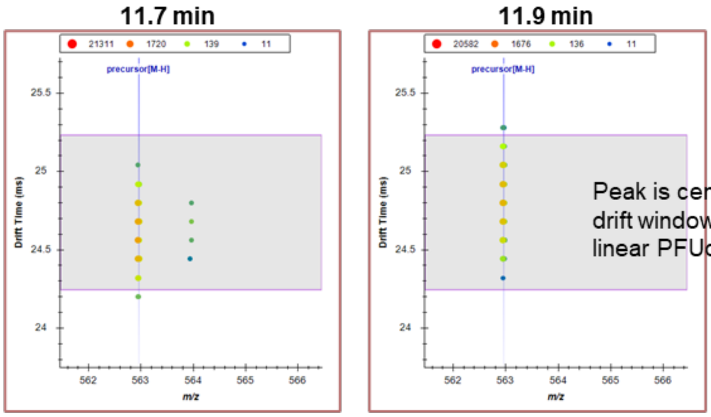

Peak is centered within the drift window, confirming linear PFUdA

C) Nested drift spectra of $M\text{-}H\text{-}CO_2$- with drift window drawn using the CCS for l-PFUdA

The center of the peak is slightly *higher* than the linear, indicating a conformation change when the CO_2 group is lost

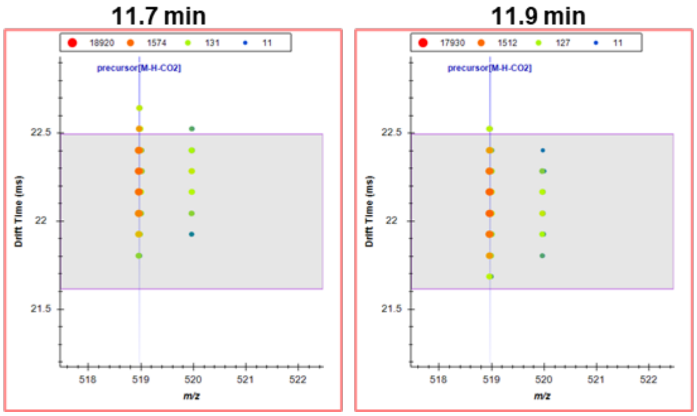

Figure S7. MS1-only data supporting co-br-PFUdA.

A) Extracted ion chromatogram (EIC) from a representative sample showing the M-H- and M-H-CO2- ions detected for both linear PFUdA and the isomer. **B)** Nested drift spectra for the M-H- ion. C**)** Nested drift spectra for the M-H-CO2- ion.

Identification and experimental measurements

- **Name:** co-br-PFUdA
- **PubChem CID:** 88337375
- **Molecular formula:** $C_{11}HF_{21}O_2$
- **Observed adduct(s):** [M-H]-, [M-H-CO_2]-
- **Exact** ***m/z*****(s):** 562.9568, 518.9670
- **Measured CCS:** 181.61 $Å^2$, 164.83 $Å^2$
- **Measured RT:** 11.70 min

Most likely candidate structure

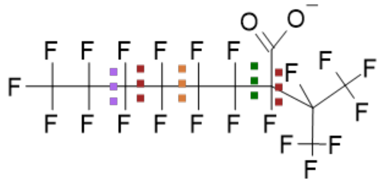

Supporting evidence

- **Mobility-aligned fragments:** Several fragments are present suggesting a fluorocarbon chain of up to 7 carbons; however abundances are quite low and can't unequivocally confirm the location of the CO2 group or the branching
- **Type of diagnostic evidence:** presence of l-PFUdA; patent information for PFUdA structural isomers
- **Predicted CCS:** The M-H- CCS is smaller than linear (182.9$Å^2$) which is expected for more compact branched molecules. However, the M-H-CO2- CCS is slightly larger than linear PFUdA's (163.4 $Å^2$), which suggests the molecule becomes more extended with a loss of CO_2
- **Predicted RT:** Earlier RT than linear expected due to shorter alkyl chain available to interact with the C18 stationary phase

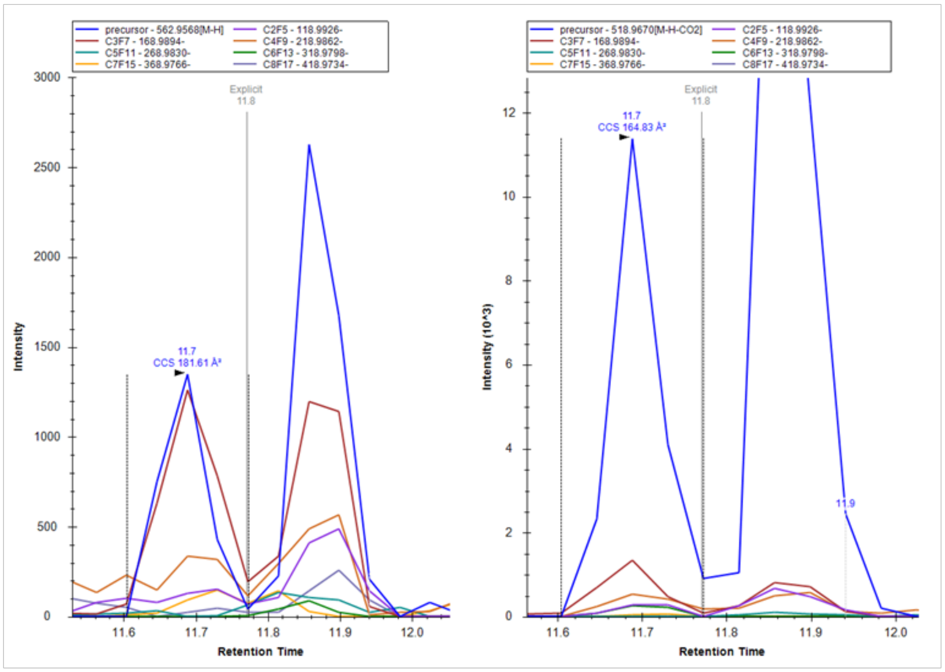

Level 3 checklist

☒ One or more structures
☒ Exact *m/z* and isotopic pattern match candidate structure
☒ CCS supported by library or predicted value
☒ RT supported by library or predicted value
☒ One or both support all candidates:
- ☒ Mobility-aligned fragments
- ☒ Diagnostic evidence

Figure S8. Supporting evidence for co-br-PFUdA.

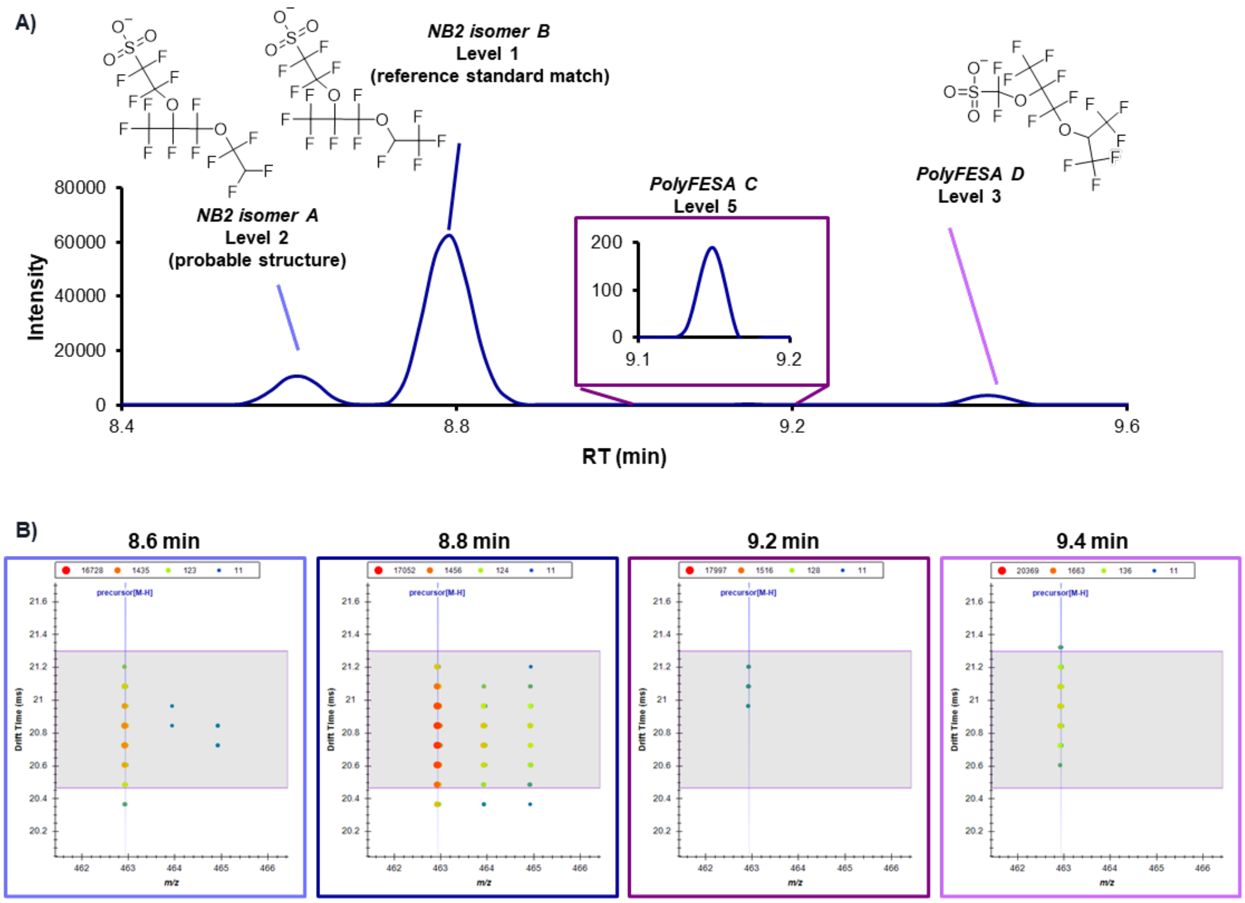

Figure S9. MS1-only data supporting polyfluoroether sulfonic acid (PolyFESA) isomers.

A) Extracted ion chromatogram (EIC) from a representative sample showing peaks for all 4 detected isomers, including two known Nafion Byproduct 2 (NB2) isomers. **B)** Nested drift spectra for each LC peak.

Identification and experimental measurements

- **Name:** Nafion Byproduct 2 (isomer A)
- **PubChem CID:** 138395264
- **Molecular formula:** $C_7H_2F_{14}O_5S$
- **Observed adduct(s):** M-H-
- **Exact *m/z*(s):** 462.9327
- **Measured CCS:** 154.04 Å^2
- **Measured RT:** 8.6 min

Probable structure

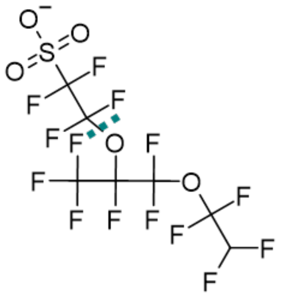

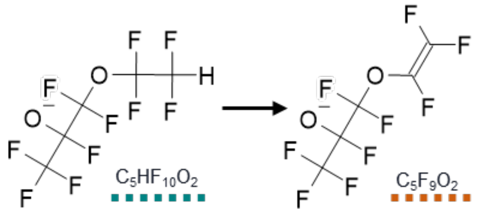

Level 2 checklist

☒ Exactly one structure
☒ Exact *m/z* and isotopic pattern match structure
☒ CCS matches library or predicted value
☒ RT matches library or predicted value
☒ One or both of:
  ☒ Mobility-aligned fragments
  ☒ Diagnostic evidence

References
(1) McCord, J. & Strynar, M. *Environmental Science & Technology* **2019** *53* (9), 4717-4727. DOI: 10.1021/acs.est.8b06017

Supporting evidence

- **Mobility-aligned fragments:** 262.9729 ($C_5F_9O_2$), 212.9779 ($C_4F_7O_2$), 82.9609 (SO_2F), 98.9558 (SO_3F), 282.9682 ($C_5F_{10}O_2H$)
- **Type of diagnostic evidence:** isomer previously reported in water (McCord & Strynar 2019, Compound 34[1]), known point source (Chemours)
- **Predicted CCS:** No literature values available but structure is expected to look very similar to isomer B (reference standard value of 155.2 Å^2)
- **Predicted RT:** Literature[1] reports eluting slightly earlier than the reference standard (8.8 min)

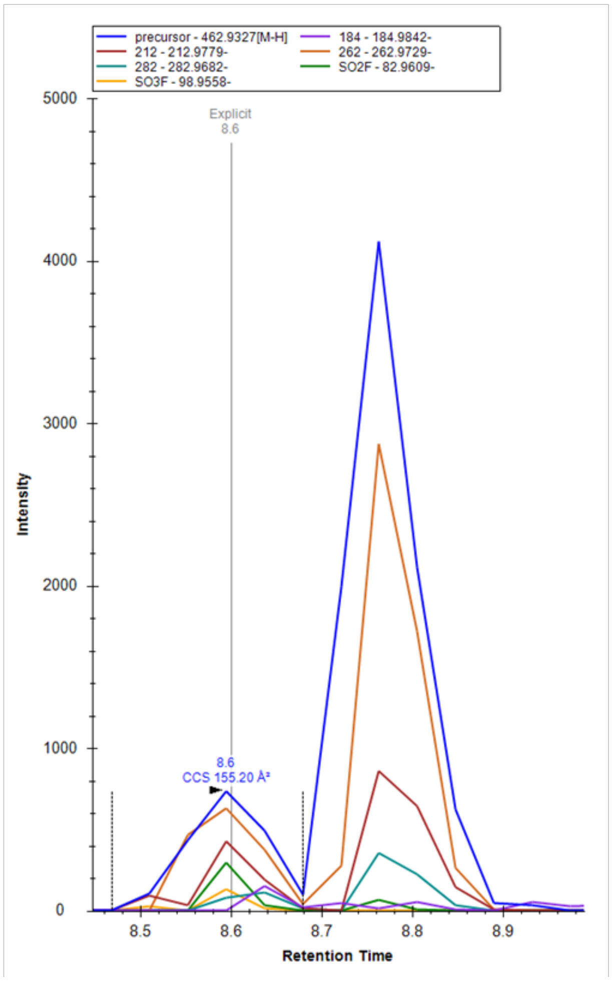

Figure S10. Supporting evidence for NB2 isomer A.

Identification and experimental measurements

- **Name:** Nafion Byproduct 2 (isomer B)
- **PubChem CID:** 14317645
- **Molecular formula:** $C_7H_2F_{14}O_5S$
- **Observed adduct(s):** M-H-
- **Exact *m/z*(s):** 462.9327
- **Measured CCS:** 154.04 $Å^2$
- **Measured RT:** 8.8 min

Supporting evidence

- **Reference standard:** Baker Lab library
- **Mobility-aligned fragments:** 262.9729 ($C_5F_9O_2$), 212.9779 ($C_4F_7O_2$), 82.9609 (SO_2F), 98.9558 (SO_3F), 282.9682 ($C_5F_{10}O_2H$)
- **Type of diagnostic evidence:** known point source (Chemours)
- **Reference CCS:** 155.2 $Å^2$
- **Reference RT:** 8.46 min

Confirmed structure

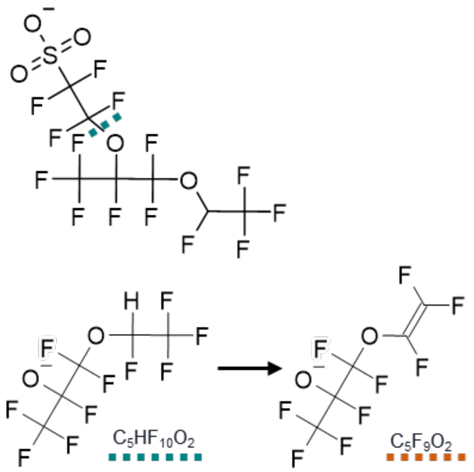

Level 1 checklist

- ☒ Exactly one structure
- ☒ Ref std analyzed under identical conditions
- ☒ Exact *m/z* and isotopic pattern match ref std
- ☒ CCS matches ref std
- ☒ RT matches ref std
- ☒ One or both of:
  - ☒ Mobility-aligned fragments
  - ☒ Diagnostic evidence

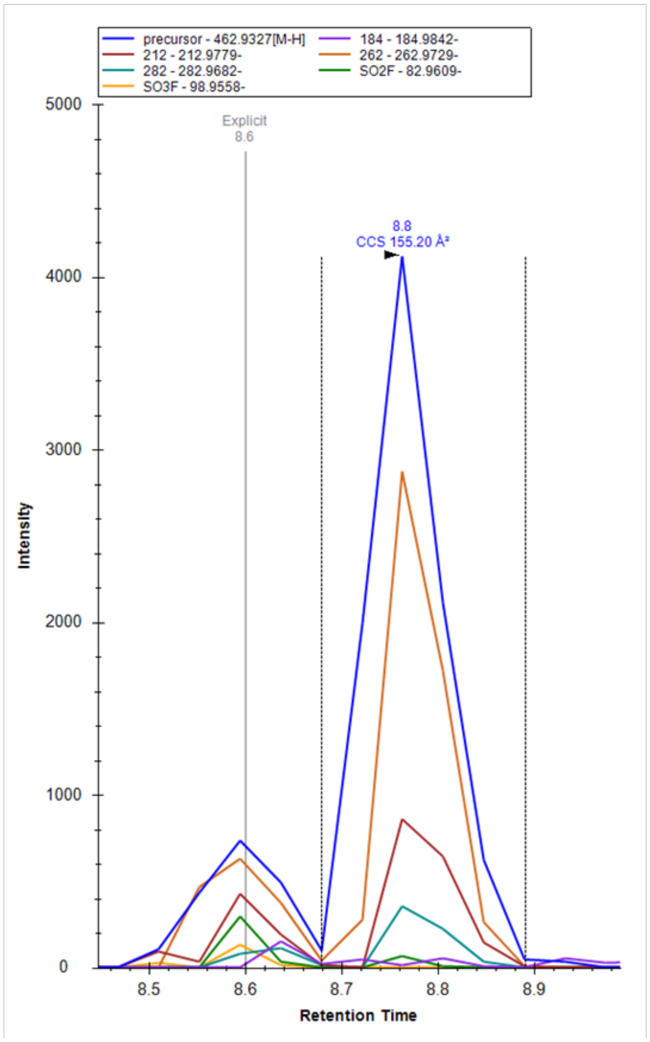

Figure S11. Supporting evidence for NB2 isomer B.

Identification and experimental measurements

- **Name:** Unknown PolyFESA C
- **PubChem CID:** n/a
- **Molecular formula:** $C_7H_2F_{14}O_5S$
- **Observed adduct(s):** M-H-
- **Exact *m/z*(s):** 462.9327
- **Measured CCS:** 156.6 Å^2
- **Measured RT:** 9.2 min

Supporting evidence

- **Mobility-aligned fragments:** none observed, insufficient precursor signal
- **Type of diagnostic evidence:** presence of other PolyFESA isomers (NB2 A and B, PolyFESA D)
- **CCS:** same CCS as PolyFESA D
- **RT:** elutes between known NB2 isomers and PolyFESA D

Originally annotated as "Nafion Byproduct 2 isomer C" but insufficient evidence available to propose a structure, therefore annotation is Level 5 until more evidence is obtained

Level 5 checklist

☒ One or more of:

- ☐ Suspect list *m/z* match
- ☒ F-like mass defect
- ☒ CCS vs *m/z*
- ☒ Other

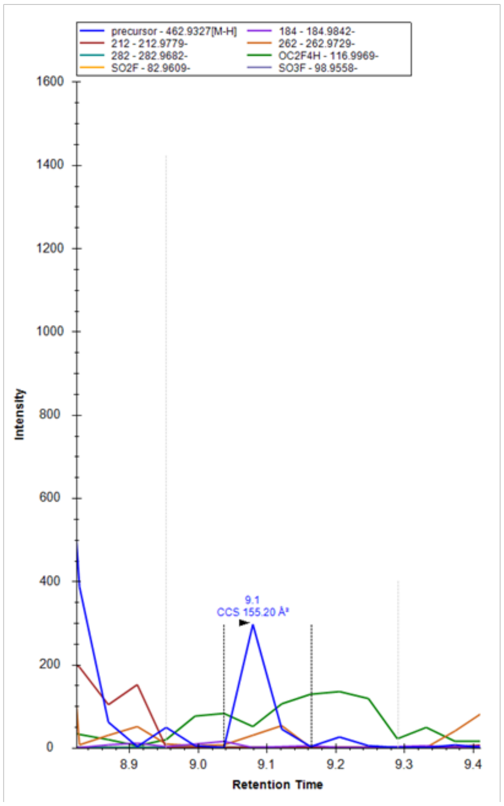

Figure S12. Supporting evidence for Unknown PolyFESA C.

Identification and experimental measurements

- **Name:** PolyFESA D
- **PubChem CID:** 57638404 (tentative)
- **Molecular formula:** $C_7H_2F_{14}O_5S$
- **Observed adduct(s):** M-H-
- **Exact *m/z*(s):** 462.9327
- **Measured CCS:** 156.6 Å^2
- **Measured RT:** 9.4 min

Supporting evidence

- **Mobility-aligned fragments:** 262.9729 ($C_5F_9O_2$), others very low abundance
- **Library CCS:** NB2 isomer B library entry CCS (155.2 Å^2)
- **Predicted RT:** elutes slightly later than other detected NB2 isomers, expected given additional CF2 on alkyl tail

Tentative candidate

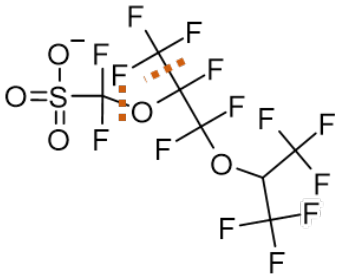

Originally annotated as "Nafion Byproduct 2 isomer D"

Level 3 checklist

☒ One or more structures
☒ Exact *m/z* and isotopic pattern match candidate structure
☒ CCS supported by library or predicted value
☒ RT supported by library or predicted value
☒ One or both support all candidates:
  ☒ Mobility-aligned fragments
  ❑ Diagnostic evidence

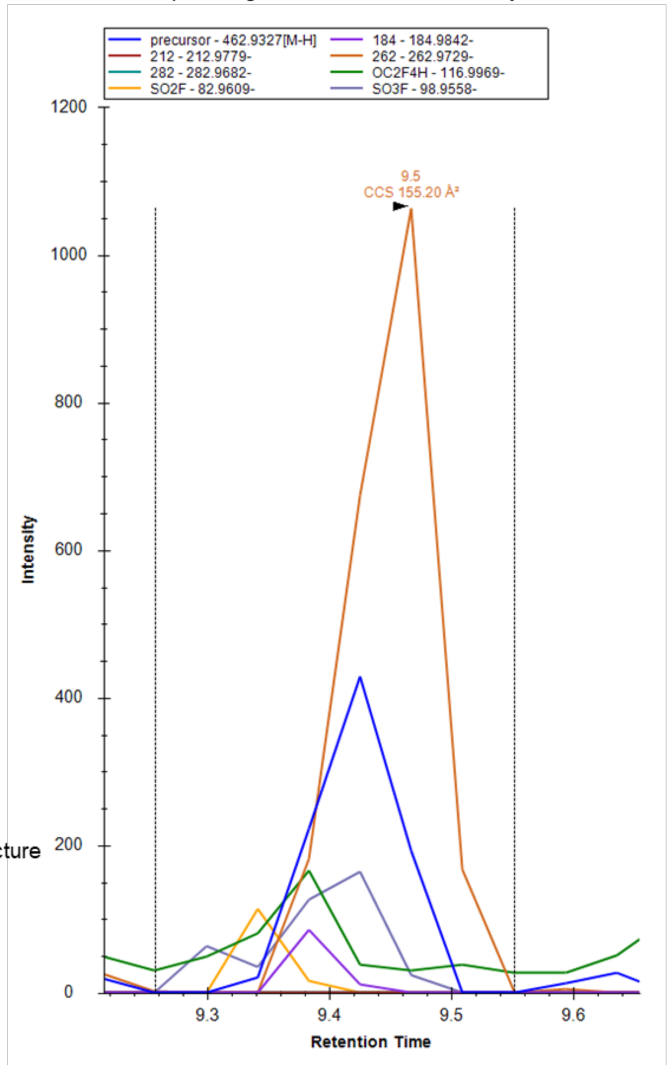

Figure S13. Supporting evidence for PolyFESA D.

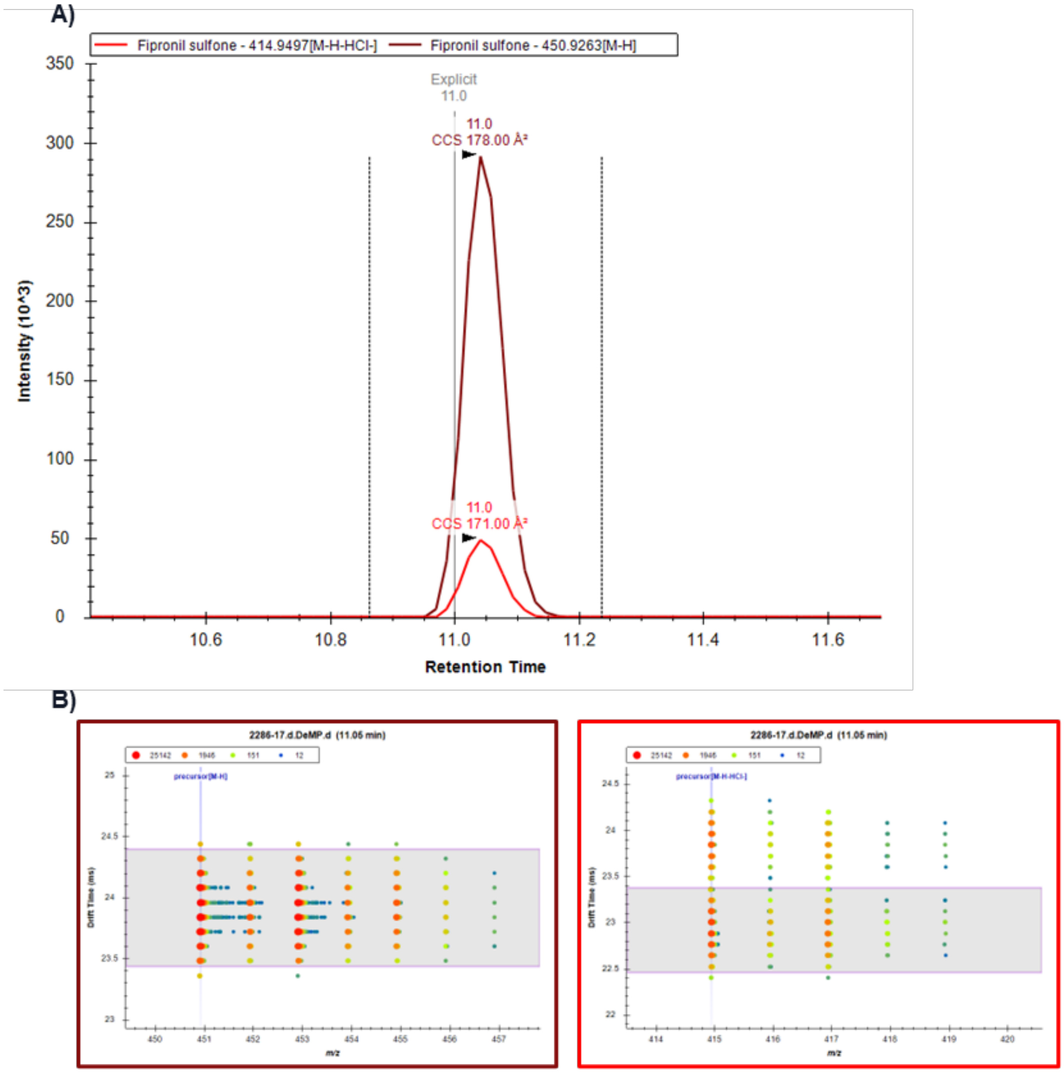

Figure S14. MS1-only data supporting fipronil sulfone.

A) Extracted ion chromatogram (EIC) from a representative sample showing peaks for both M-H- and M-H-HCl- isomers. **B)** Nested drift spectra for both ions.

Identification and experimental measurements

- **Name:** Fipronil sulfone
- **PubChem CID:** 3078139
- **Molecular formula:** $C_{12}H_4Cl_2F_6N_4O_2S$
- **Observed adduct(s):** M-H-, M-H-HCl-
- **Exact *m/z*(s):** 450.9263, 414.9497
- **Measured CCS:** 177.42 Å^2, 170.72 Å^2
- **Measured RT:** 11.0 min

Probable structure

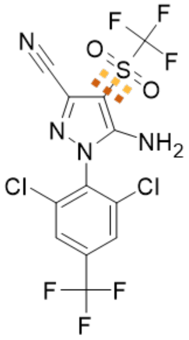

Level 2 checklist

☒ Exactly one structure
☒ Exact *m/z* and isotopic pattern match structure
☒ CCS matches library or predicted value
☒ RT matches library or predicted value
☒ One or both of:
  ☒ Mobility-aligned fragments
  ❑ Diagnostic evidence

References

(1) Weed, R.A.; Campbell, G.; Brown, L.; May, K.; Sargent, D.; Sutton, E.; Burdette, K.; Rider, W.; Baker, E.S.; Enders, J.R. Non-Targeted PFAS Suspect Screening and Quantification of Drinking Water Samples Collected through Community Engaged Research in North Carolina's Cape Fear River Basin. *Toxics* **2024**, *12*, 403. https://doi.org/10.3390/toxics12060403

Supporting evidence

- **Mobility-aligned fragments:** match to spectrum reported by Weed *et al*. 2024[1]
- **Predicted CCS: no library values available in ESI(-)**
- **Predicted RT:** select one and add

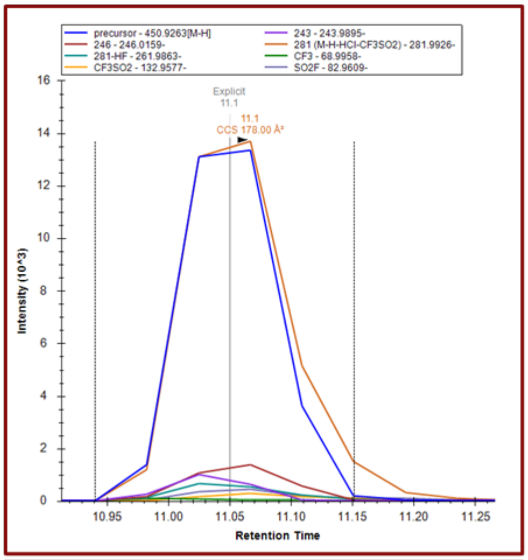

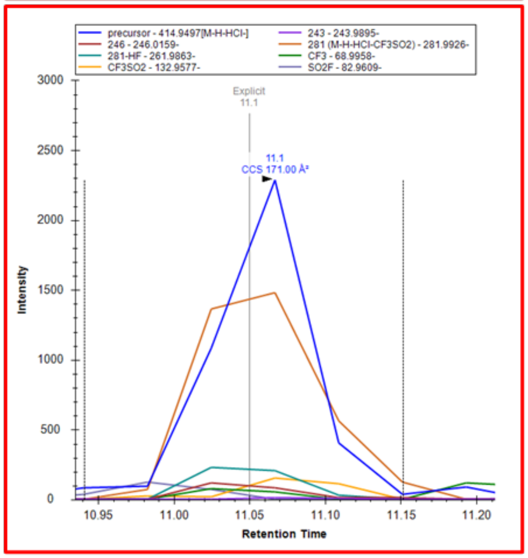

Figure S15. Supporting evidence for fipronil sulfone.

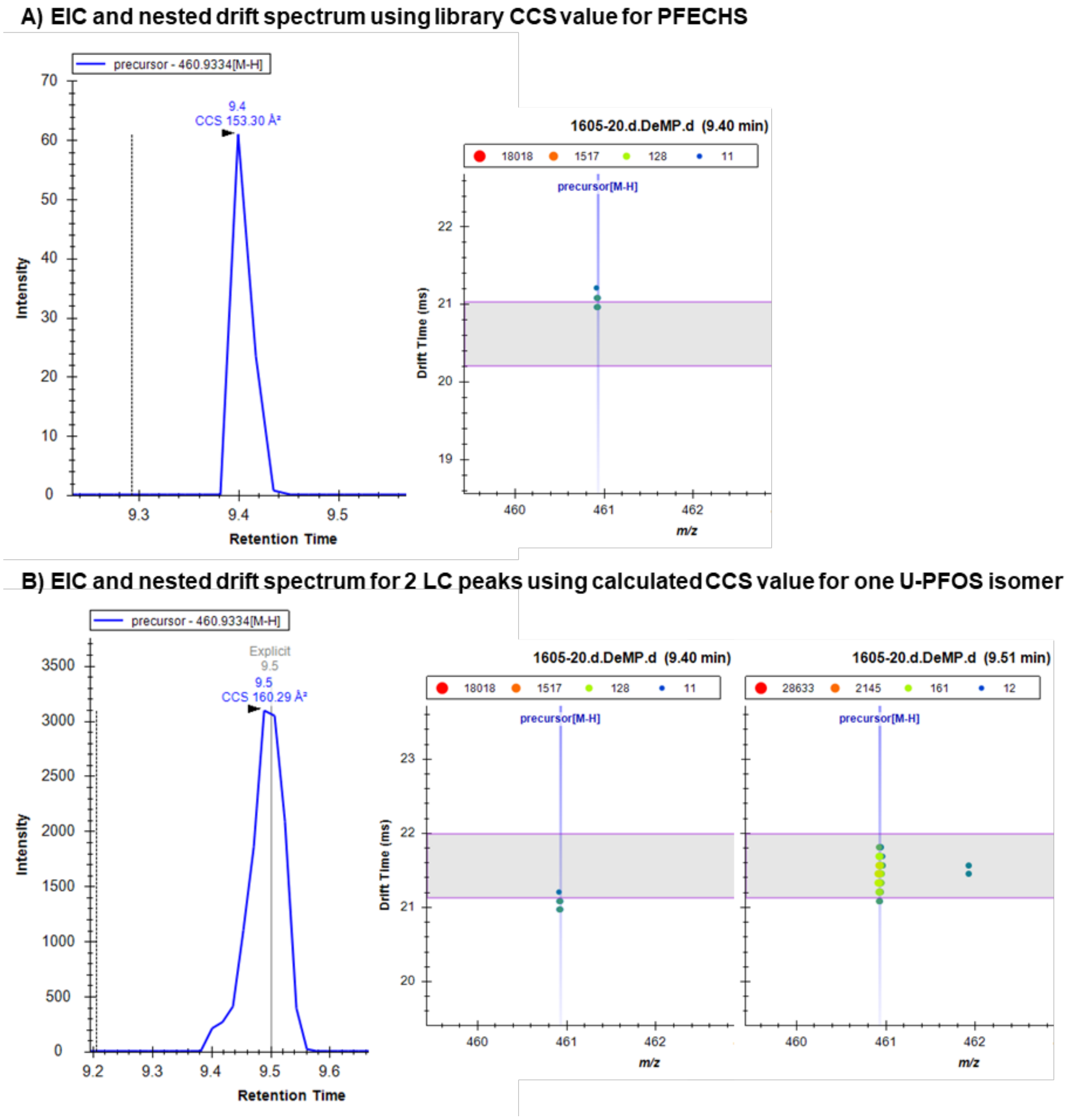

Figure S16. MS1-only data supporting U-PFOS.

A) Extracted ion chromatogram (EIC) and nested drift spectrum using library CCS values for PFECHS, a structural isomer of U-PFOS. Horizontal bars show the drift window for PFECHS using a resolving power of 50. **B)** EIC using the calculated CCS for one U-PFOS isomer and drift spectra from two unresolved points along the LC peak showing presence of at least 2 isomers. The peak at 9.40 min is integrated in both A and B but clearly has a different CCS than either PFECHS or the isomer at 9.51 min.

Identification and experimental measurements

- **Name:** U-PFOS
- **PubChem CID:** n/a
- **Molecular formula:** $C_8HF_{15}O_3S$
- **Observed adduct(s):** M-H-
- **Exact *m/z*(s):** 460.9334
- **Measured CCS:** 155-160 Å^2
- **Measured RT:** 9.4-9.5 min (multiple peaks)

Tentative candidates

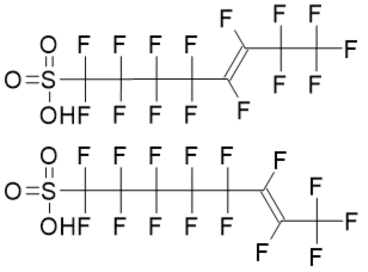

Level 3 checklist

☒ One or more structures
☒ Exact *m/z* and isotopic pattern match candidate structure
☒ CCS supported by library or predicted value
☒ RT supported by library or predicted value
☒ One or both support all candidates:
  ☐ Mobility-aligned fragments
  ☒ Diagnostic evidence

References

(1) Kirkwood-Donelson K. I., Dodds, J. N., Schnetzer, A., Hall, N., Baker, E. S. Uncovering per- and polyfluoroalkyl substances (PFAS) with nontargeted ion mobility spectrometry–mass spectrometry analyses. Sci. Adv. 9 (2023). DOI: 10.1126/sciadv.adj7048

Supporting evidence

- **Mobility-aligned fragments:** inconclusive, several seen but low abundance and not diagnostic
- **Type of diagnostic evidence:** Manufacturing knowledge; known point source (Chemours), prior detections in water[1]
- **Library CCS:** Previously reported by Donelson *et al.* 2023[1] as larger than library PFECHS (153.3 Å^2)
- **Predicted RT:** expected to elute later than library PFECHS (9.0 min) due to longer fluoroalkyl tail; also seen in prior detections[1]

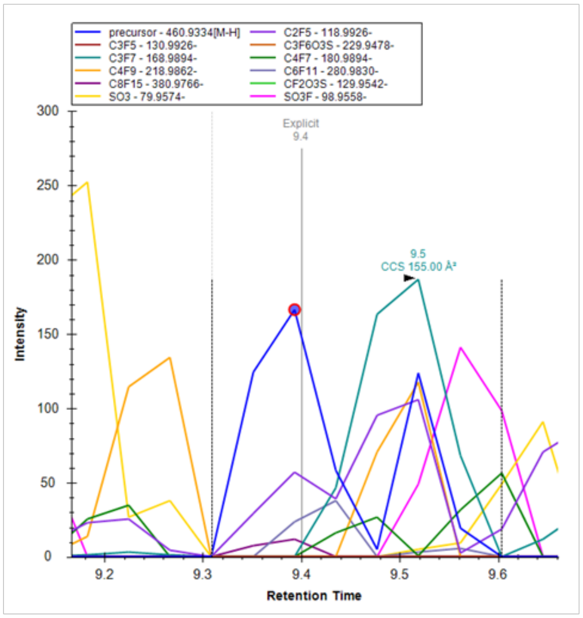

Figure S17. Supporting evidence for U-PFOS.

Identification and experimental measurements

- **Name:** PFPeDA
- **PubChem CID:** 12731198
- **Molecular formula:** $C_{15}HF_{29}O_2$
- **Observed adduct(s):** M-H-, M-H-CO_2-
- **Exact *m/z*(s):** 762.9441, 718.9542
- **Measured CCS:** 218.70 Å^2, 197.00 Å^2
- **Measured RT:** 13.8 min

Supporting evidence

- **Mobility-aligned fragments:** signal intensity low, none observed
- **Type of diagnostic evidence:** homologous series present in sample set
- **Library CCS:** Literature value reported by Boatman *et al.* 2024[1]
- **Library RT:** Literature value reported by Boatman *et al.* 2024[1] using identical method

Probable structure

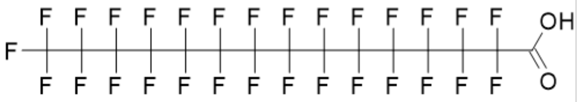

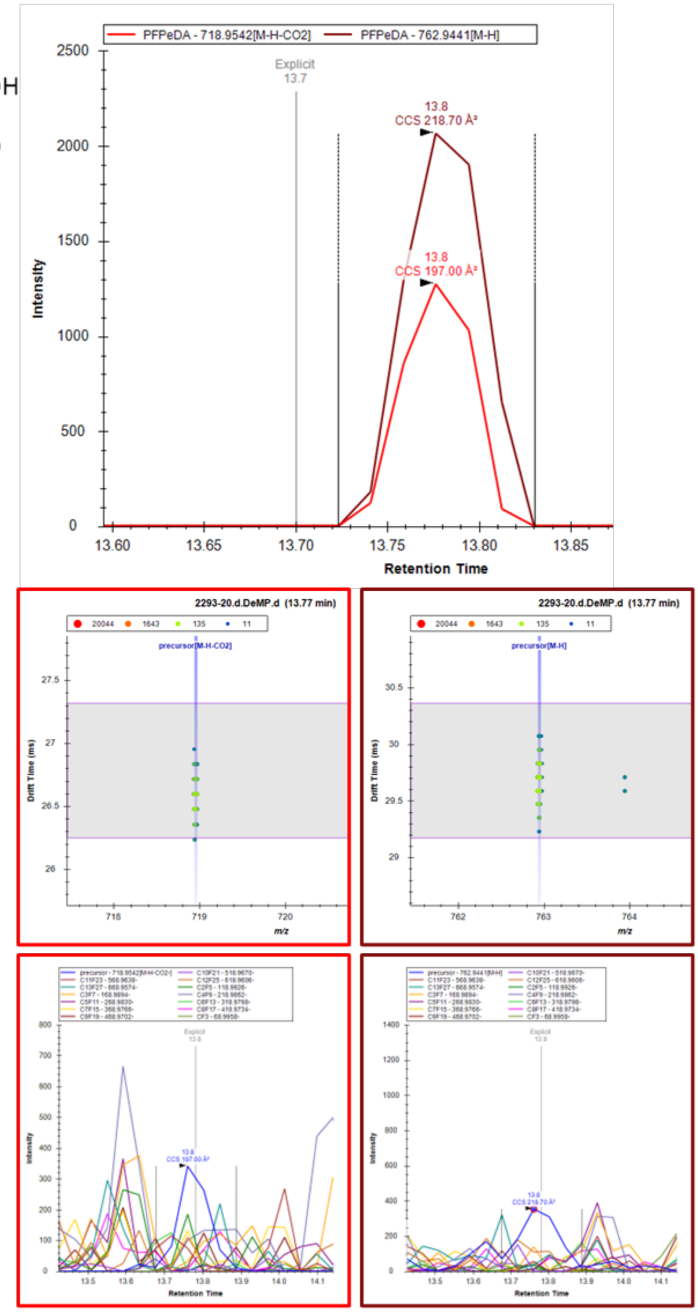

Level 2 checklist

☒ Exactly one structure
☒ Exact *m/z* and isotopic pattern match structure
☒ CCS matches library or predicted value
☒ RT matches library or predicted value
☒ One or both of:
  - ☐ Mobility-aligned fragments
  - ☒ Diagnostic evidence

References

(1) Anna K. Boatman, Jessie R. Chappel, Madison E. Polera, James N. Dodds, Scott M. Belcher, and Erin S. Baker. Assessing Per- and Polyfluoroalkyl Substances in Fish Fillet Using Non-Targeted Analyses. *Environmental Science & Technology* **2024** *58* (32), 14486-14495 DOI: 10.1021/acs.est.4c04299

Figure S18. Supporting evidence for PFPeDA.

Identification and experimental measurements

- **Name:** PFUdS
- **PubChem CID:** 22141518
- **Molecular formula:** $C_{11}HF_{23}O_3S$
- **Observed adduct(s):** M-H-
- **Exact *m/z*(s):** 648.9206
- **Measured CCS:** br: 191.8 Å^2, l: 195.13 Å^2
- **Measured RT:** br: 12.2 min, l: 12.4 min

Supporting evidence

- **Mobility-aligned fragments:** low signal intensity, low abundance SO_3- fragment possibly observed
- **Type of diagnostic evidence:** homologous series in sample set
- **Library CCS:** Literature values (br: 191.77 Å^2, l: 194.27 Å^2) reported by Boatman *et al*. 2024[1]
- **Library RT:** Literature value (12.3 min) reported by Boatman *et al*. 2024[1] using identical method

Probable structure*

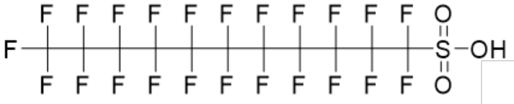

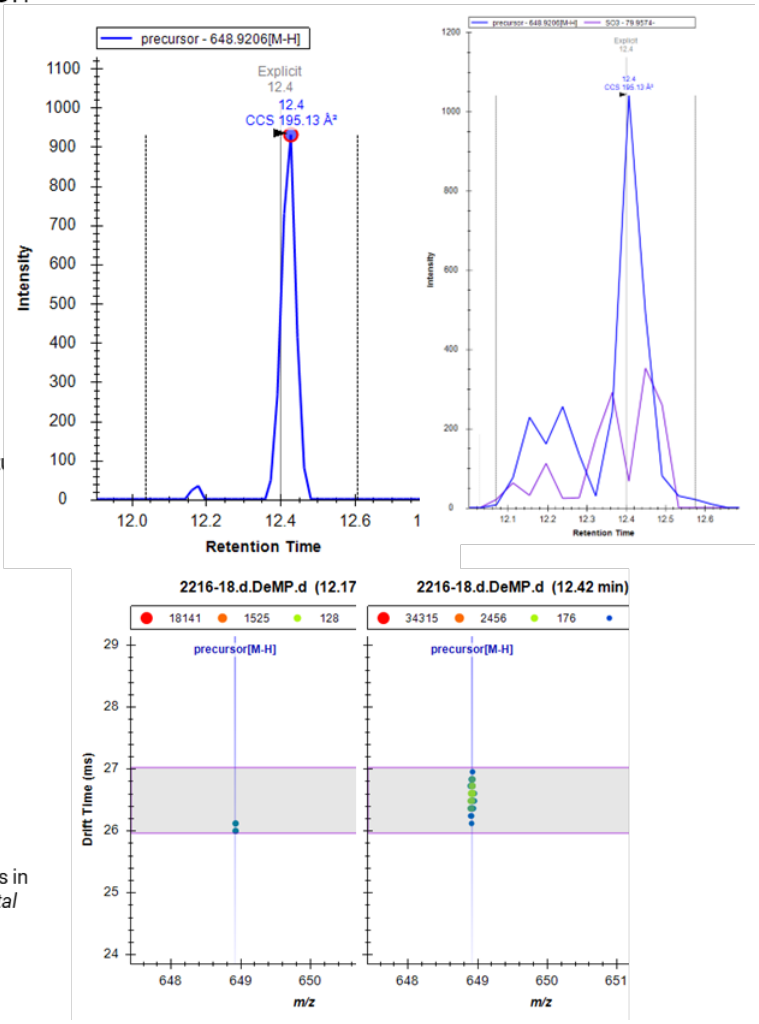

*Branched isomer also detected at Level 3 confidence (undetermined branch location)

Level 2 checklist

☒ Exactly one structure
☒ Exact *m/z* and isotopic pattern match structu
☒ CCS matches library or predicted value
☒ RT matches library or predicted value
☒ One or both of:
  ❑ Mobility-aligned fragments
  ☒ Diagnostic evidence

References

(1) Anna K. Boatman, Jessie R. Chappel, Madison E. Polera, James N. Dodds, Scott M. Belcher, and Erin S. Baker. Assessing Per- and Polyfluoroalkyl Substances in Fish Fillet Using Non-Targeted Analyses. *Environmental Science & Technology* **2024** *58* (32), 14486-14495 DOI: 10.1021/acs.est.4c04299

Figure S19. Supporting evidence for PFUdS.

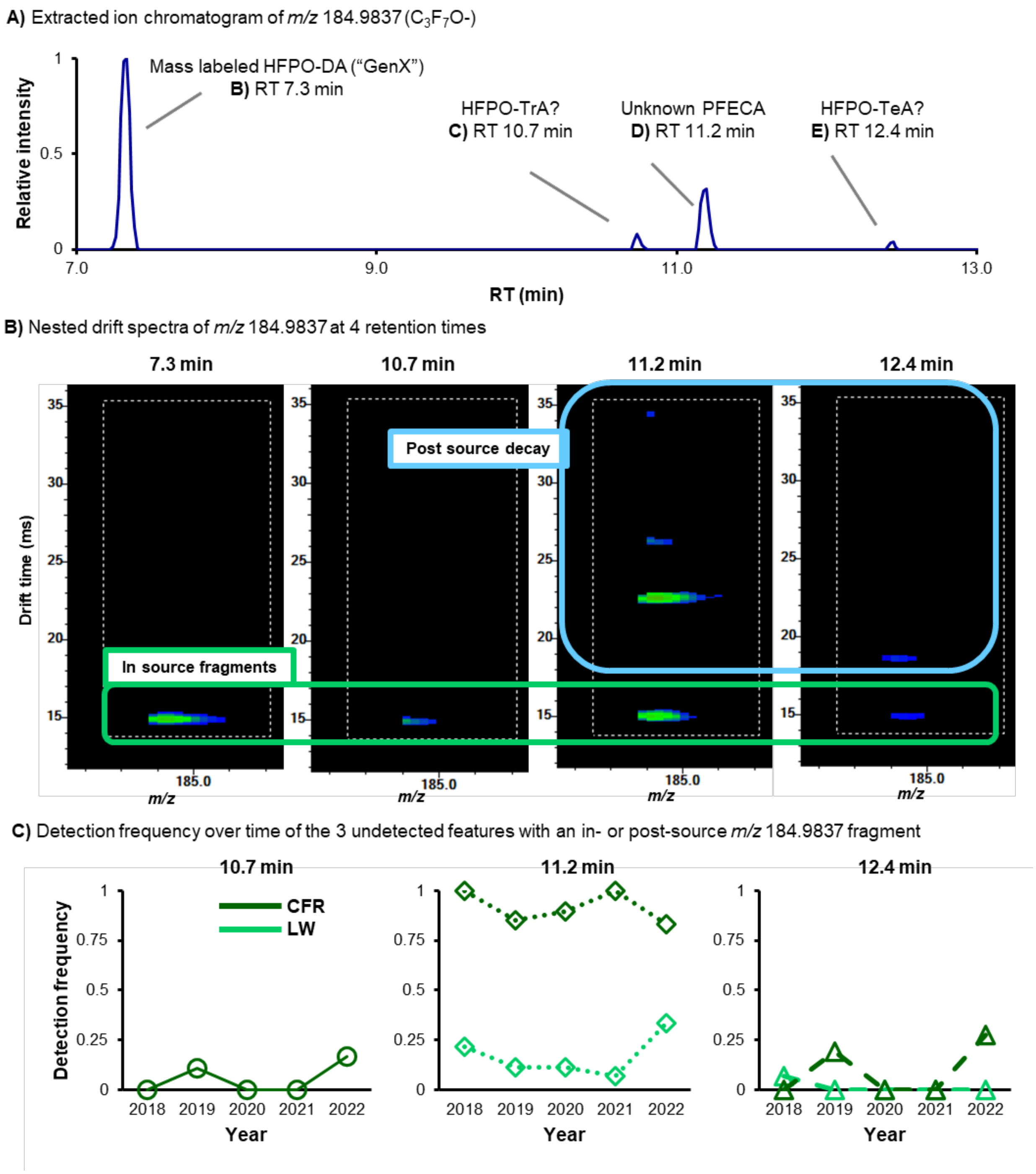

Figure S20. MS1-only fragmentation evidence of undetected PFECAs.
A) EIC of C3F7O peaks **B)** Nested drift spectra. **C)** Detection frequency trends over time.

Supplemental Relative Abundance Evaluation

Our primary interest in this non-targeted study was detecting and identifying as many different PFAS as possible, and our discovery workflow allowed us to identify 5 previously unreported PFAS (Compound 592, Compound 628, PFHxESA, PFHpESA, and co-br-PFUdA). While absolute quantification of these analytes is not possible without standards, we performed a preliminary assessment of spatial and temporal trends by comparing the detection frequency and relative abundance of each analyte of interest. Detection frequency is the number of alligators with the molecule detected divided by total number of alligators tested. Relative abundance (here defined as the total peak area of the analyte normalized to the peak area of a surrogate standard) is correlated with concentration, but without standards we cannot accurately fit a calibration curve to quantify these molecules. To minimize the effects of covariates, we selected the two sites with all 5 years of data available for the temporal trend analysis (Greenfield Lake in the Cape Fear River (CFR) and the reference site, Lake Waccamaw (LW)). For the spatial trend analysis, we selected all sites with $n \geq 3$ alligators from 2019 (the year with the most data available from different sites), including GL, LW, and 3 additional sites within the CFR: Orton Pond (OP), Oak Island (OI), and Bald Head Island (BI). None of the novel compounds were detected in FL alligators, which supports the North Carolina Chemours facility as a potential point source.

Figure S21 shows detection frequency and average relative abundances for the 5 novel PFAS reported in biota here for the first time, with the figures in the left side panel showing temporal trends and the figures on the right showing site trends. Looking first at the temporal trends in GL and LW, the detection frequencies are not changing over time. In general, detection frequencies of all 5 novel PFAS were much lower in the LW alligators vs GL alligators for all 5 years, which supports lower levels of exposure at the reference site. The relative abundances were also generally lower in LW vs GL alligators over all 5 years, but interestingly in 2018 the average relative abundances of Compound 592 and Compound 628 were as high or higher than the GL alligators. These results suggest that the GL alligators have ongoing exposure to these 5

novel PFAS, while the LW alligators' exposure may have lessened over the 5 year time span. Nearly 100% of GL alligators had 4 of the novel PFAS (all except Compound 592) detected each year, and the relative abundances were not changing year to year. The detection frequency of Compound 592 was lower, less than 50% all years, and had more variable relative abundance, with no clear time course trends. While no biological half-life data is available these novel PFAS, work by Dodds et al.[1] indicated that the levels of related compounds (Compound 10[2], the shorter chain PFAESAs, and l-PFUdA) in the CFR significantly decreased between 2017 and 2022. Our findings suggest that the CFR gators have ongoing exposure, possibly from manufacturing waste, accumulation in sediment, or consumption of contaminated food.

Looking next at spatial trends, we expected to see higher detection frequencies and relative abundances in the upstream alligators which are closer to the Chemours manufacturing facility. PFHxESA followed the expected trend: the highest relative abundances were seen in the GL alligators at nearly 100% detection frequency, followed by BI and OI alligators, and finally OP, with no detections in LW in 2019. PFHpESA and co-br-PFUdA were only detected in GL alligators in 2019, also at nearly 100% detection frequency. The OP site is geographically closer to Chemours but is not directly downstream, which could explain the lower levels in these alligators. It is possible that these molecules were present but not at high enough levels to be detectable in gators at other sites. Interestingly, however, both novel triethers (Compounds 592 and 628) had several orders of magnitude higher relative abundance in the alligators from BI and OI compared to the upstream sites GL and OP in 2019. These findings suggest that the novel triether PFAS are highly mobile and bioaccumulative, or possibly that different microbial or internal metabolic pathways are impacting novel triether exposure in the BI and OI alligators.

Peak areas for all PFAS detected in alligators were exported from Skyline (**Table S5**) and a preliminary relative abundance analysis was performed. In general, no obvious changes in abundance over time were observed. Box plots of relative abundances for each analyte broken

down by watershed and year are show in **Figures S22-S37**. The relative abundance results described here are limited and should be considered preliminary. Future work should include in-depth correlation analysis with parameters such as alligator sex and size, seasonality, specific location within each watershed, and health endpoints. However, as no toxicity data is available for any of these PFAS reported here, their presence in alligators should nevertheless be highly concerning to researchers, regulators, and residents. Ongoing monitoring and further studies are urgently needed to understand the potential risks of exposure to these novel compounds.

References

(1) Dodds, J. N.; Kirkwood-Donelson, K. I.; Boatman, A. K.; Knappe, D. R. U.; Hall, N. S.; Schnetzer, A.; Baker, E. S. Evaluating Solid Phase Adsorption Toxin Tracking (SPATT) for passive monitoring of per- and polyfluoroalkyl substances (PFAS) with Ion Mobility Spectrometry-Mass Spectrometry (IMS-MS). *Science of The Total Environment* **2024**, *947*, 174574. DOI: https://doi.org/10.1016/j.scitotenv.2024.174574.

(2) Kirkwood-Donelson, K. I.; Dodds, J. N.; Schnetzer, A.; Hall, N.; Baker, E. S. Uncovering per- and polyfluoroalkyl substances (PFAS) with nontargeted ion mobility spectrometry-mass spectrometry analyses. *Sci Adv* **2023**, *9* (43), eadj7048. DOI: 10.1126/sciadv.adj7048 From NLM.

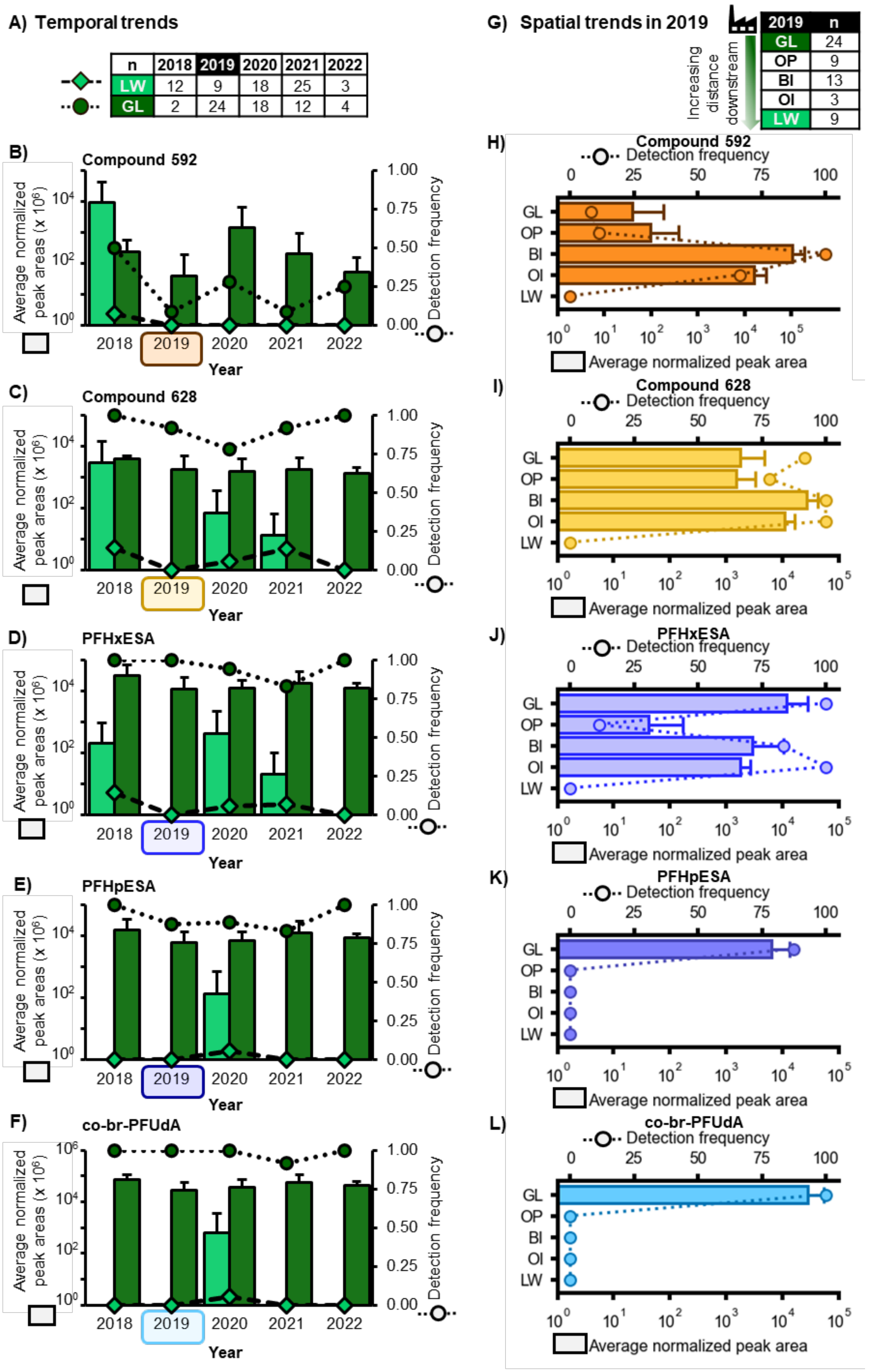

A) Temporal trends
n 2018 2019 2020 2021 2022
LW 12 9 18 25 3
GL 2 24 18 12 4
G) Spatial trends in 2019
Increasing distance downstream
2019 n
GL 24
OP 9
BI 13
OI 3
LW 9
B) Compound 592
C) Compound 628
D) PFHxESA
E) PFHpESA
F) co-br-PFUdA
Average normalized peak areas (x 10^6)
Detection frequency
Year
2018 2019 2020 2021 2022
H) Compound 592
I) Compound 628
J) PFHxESA
K) PFHpESA
L) co-br-PFUdA
Detection frequency
Average normalized peak area
GL OP BI OI LW

Figure S21. Preliminary temporal and spatial trend analysis for the 5 novel PFAS.

GL: Greenfield Lake; LW: Lake Waccamaw; OP: Orton Pond; BI: Bald Head Island; OI: Oak Island. **A)** Legend and number of alligators per year for the two sites with all 5 years of data available. **B-F)** Temporal trends in relative abundance (bar chart, left y-axis) and detection frequency (line graph with markers, right y-axis) over the 5 year study. **G)** Number of alligators per site for 2019, the year with the most different sites sampled with an n > 3 alligators per site.**H-L)** Site trends in relative abundance (bar chart, bottom axis) and detection frequency (line graph with markers, top axis). Sites are shown in order of distance downstream of Chemours.

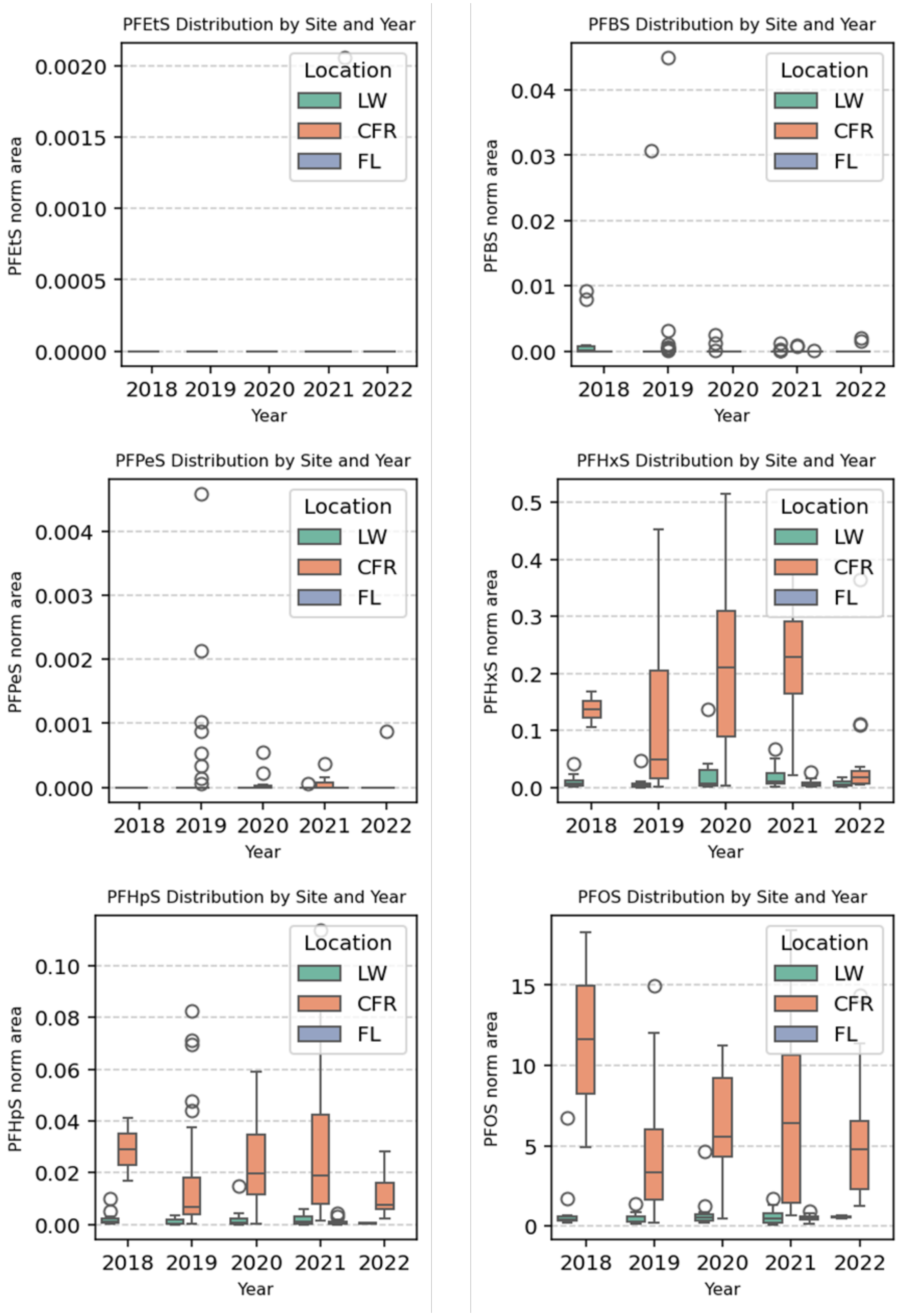
PFEtS Distribution by Site and Year
PFEtS norm area
0.0020
0.0015
0.0010
0.0005
0.0000
2018 2019 2020 2021 2022
Year
Location
LW
CFR
FL
PFBS Distribution by Site and Year
PFBS norm area
0.04
0.03
0.02
0.01
0.00
2018 2019 2020 2021 2022
Year
Location
LW
CFR
FL
PFPeS Distribution by Site and Year
PFPeS norm area
0.004
0.003
0.002
0.001
0.000
2018 2019 2020 2021 2022
Year
Location
LW
CFR
FL
PFHxS Distribution by Site and Year
PFHxS norm area
0.5
0.4
0.3
0.2
0.1
0.0
2018 2019 2020 2021 2022
Year
Location
LW
CFR
FL
PFHpS Distribution by Site and Year
PFHpS norm area
0.10
0.08
0.06
0.04
0.02
0.00
2018 2019 2020 2021 2022
Year
Location
LW
CFR
FL
PFOS Distribution by Site and Year
PFOS norm area
15
10
5
0
2018 2019 2020 2021 2022
Year
Location
LW
CFR
FL

Figure S22. Relative abundance trends for PFSAs (PFEtS through PFOS).

Box plots showing median and range of relative abundance for all detected PFSAs (PFEtS through PFOS) by watershed and year. Relative abundance is defined as the normalized peak area (total peak area divided by area of a surrogate standard)

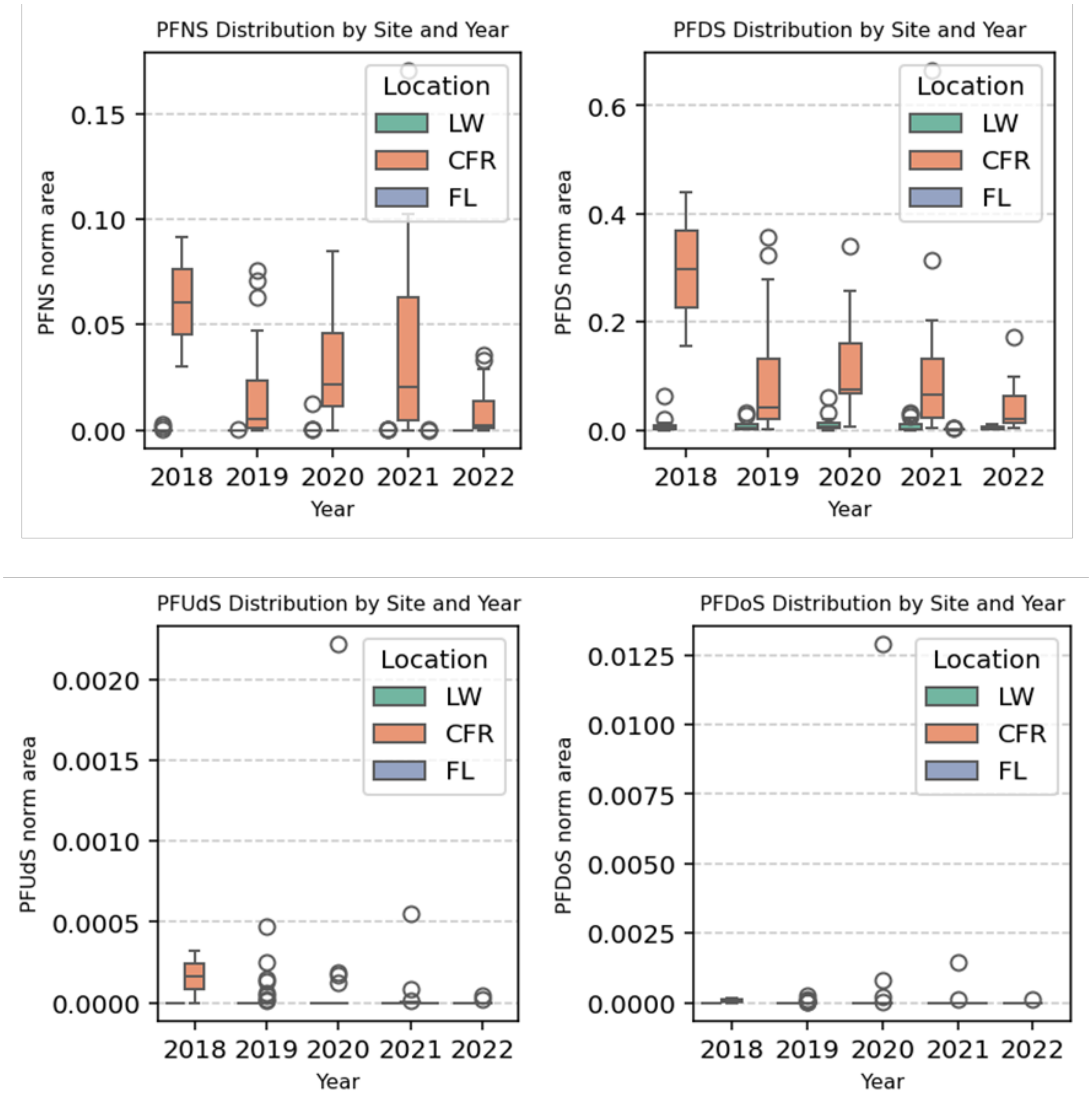

Figure S23. Relative abundance trends for PFSAs (PFNS through PFDoS).

Box plots showing median and range of relative abundance for all detected PFSAs (PFNS through PFDoS) by watershed and year. Relative abundance is defined as the normalized peak area (total peak area divided by area of a surrogate standard)

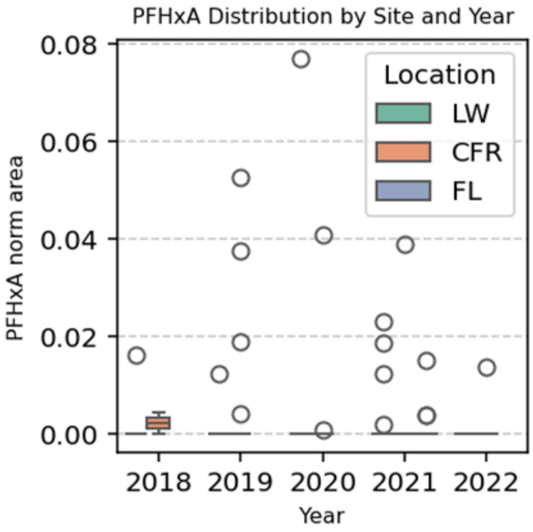
PFHxA Distribution by Site and Year
PFHxA norm area
0.08
0.06
0.04
0.02
0.00
Location
LW
CFR
FL
2018
2019
2020
2021
2022
Year

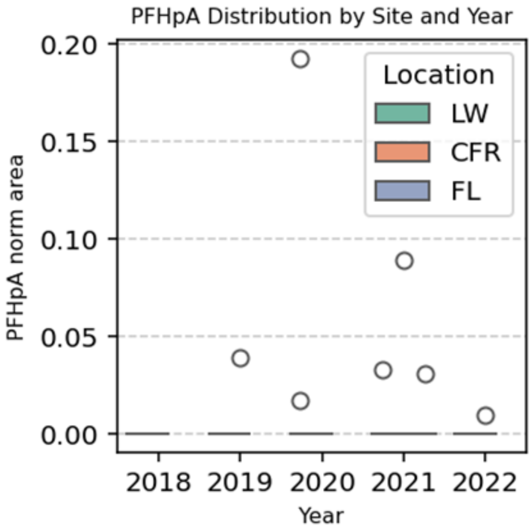
PFHpA Distribution by Site and Year
PFHpA norm area
0.20
0.15
0.10
0.05
0.00
Location
LW
CFR
FL
2018
2019
2020
2021
2022
Year

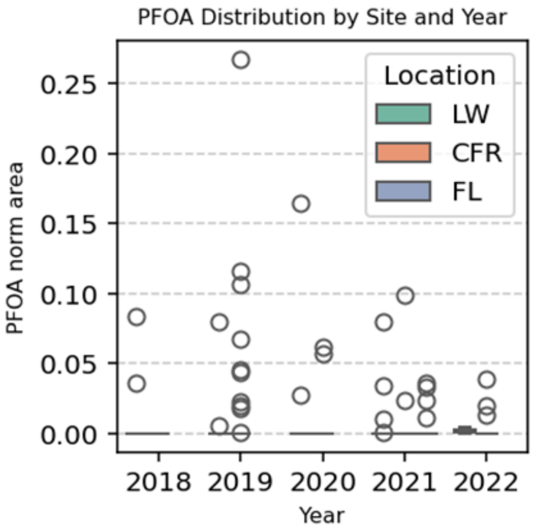
PFOA Distribution by Site and Year
PFOA norm area
0.25
0.20
0.15
0.10
0.05
0.00
Location
LW
CFR
FL
2018
2019
2020
2021
2022
Year

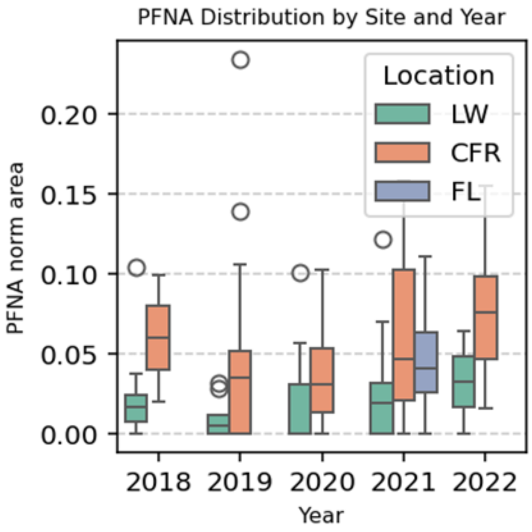
PFNA Distribution by Site and Year
PFNA norm area
0.20
0.15
0.10
0.05
0.00
Location
LW
CFR
FL
2018
2019
2020
2021
2022
Year

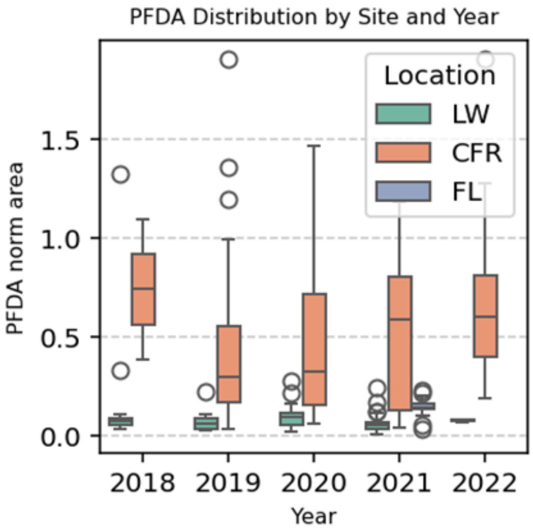
PFDA Distribution by Site and Year
PFDA norm area
1.5
1.0
0.5
0.0
Location
LW
CFR
FL
2018
2019
2020
2021
2022
Year

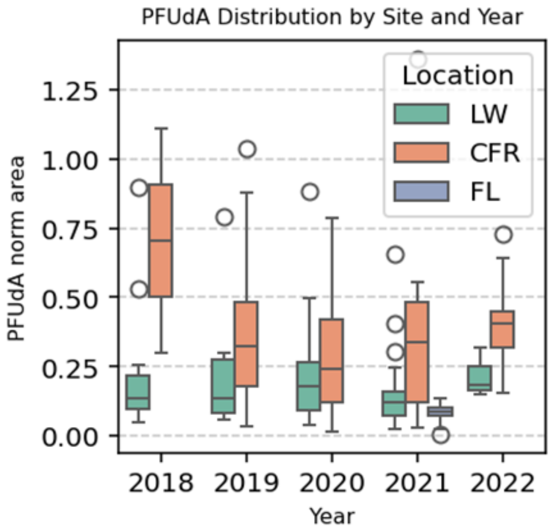
PFUdA Distribution by Site and Year
PFUdA norm area
1.25
1.00
0.75
0.50
0.25
0.00
Location
LW
CFR
FL
2018
2019
2020
2021
2022
Year

Figure S24. Relative abundance trends for PFCAs (PFHxA through PFUdA).

Box plots showing median and range of relative abundance for all detected PFCAs (PFHxA through PFUdA) by watershed and year. Relative abundance is defined as the normalized peak area (total peak area divided by area of a surrogate standard)

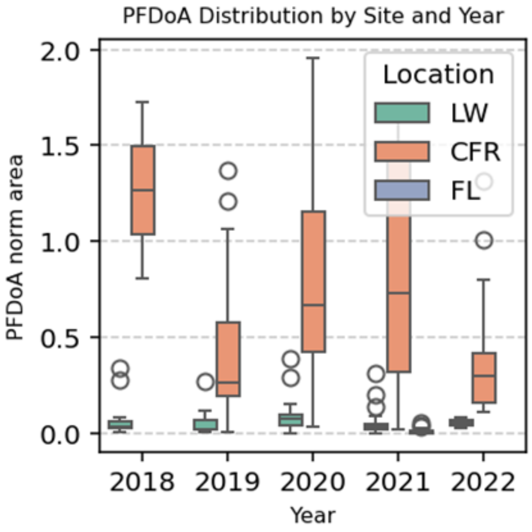
PFDoA Distribution by Site and Year
Location
LW
CFR
FL
PFDoA norm area
2.0
1.5
1.0
0.5
0.0
2018 2019 2020 2021 2022
Year

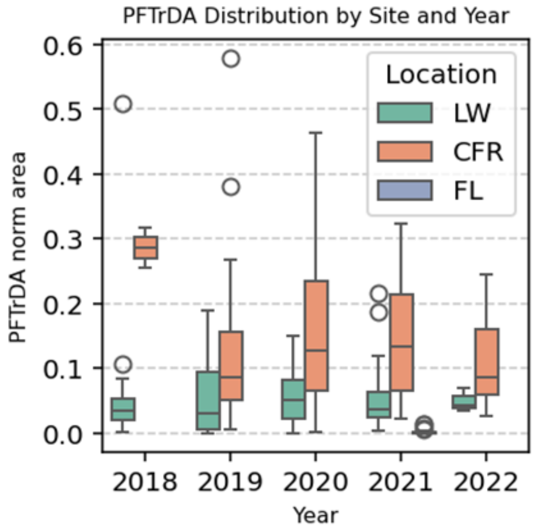
PFTrDA Distribution by Site and Year
Location
LW
CFR
FL
PFTrDA norm area
0.6
0.5
0.4
0.3
0.2
0.1
0.0
2018 2019 2020 2021 2022
Year

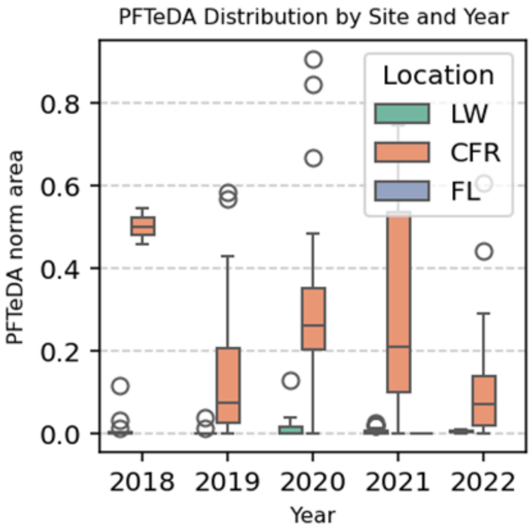
PFTeDA Distribution by Site and Year
Location
LW
CFR
FL
PFTeDA norm area
0.8
0.6
0.4
0.2
0.0
2018 2019 2020 2021 2022
Year

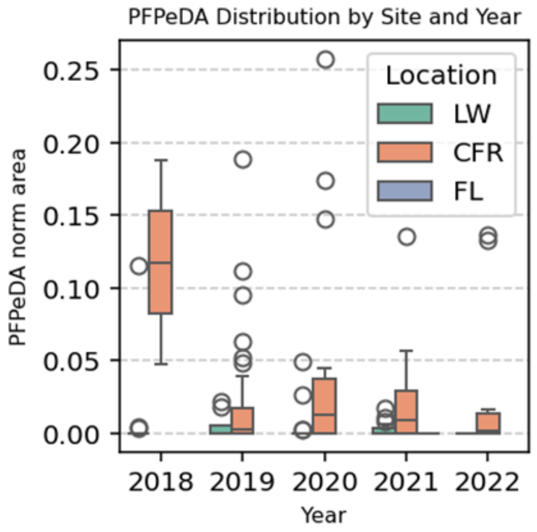
PFPeDA Distribution by Site and Year
Location
LW
CFR
FL
PFPeDA norm area
0.25
0.20
0.15
0.10
0.05
0.00
2018 2019 2020 2021 2022
Year

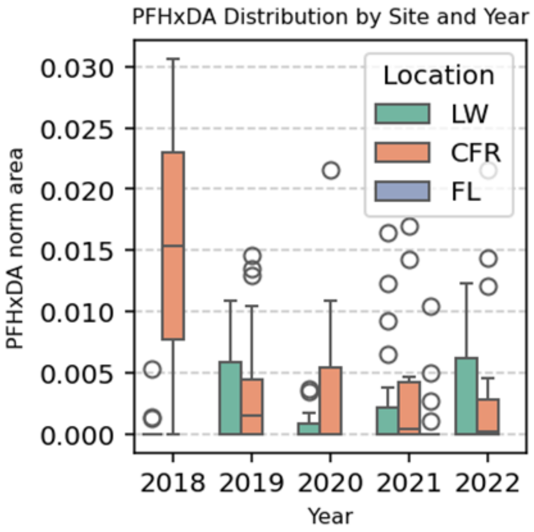
PFHxDA Distribution by Site and Year
Location
LW
CFR
FL
PFHxDA norm area
0.030
0.025
0.020
0.015
0.010
0.005
0.000
2018 2019 2020 2021 2022
Year

Figure S25. Relative abundance trends for PFCAs (PFDoA through PFHxDA).

Box plots showing median and range of relative abundance for all detected PFCAs (PFDoA through PFHxDA) by watershed and year. Relative abundance is defined as the normalized peak area (total peak area divided by area of a surrogate standard)

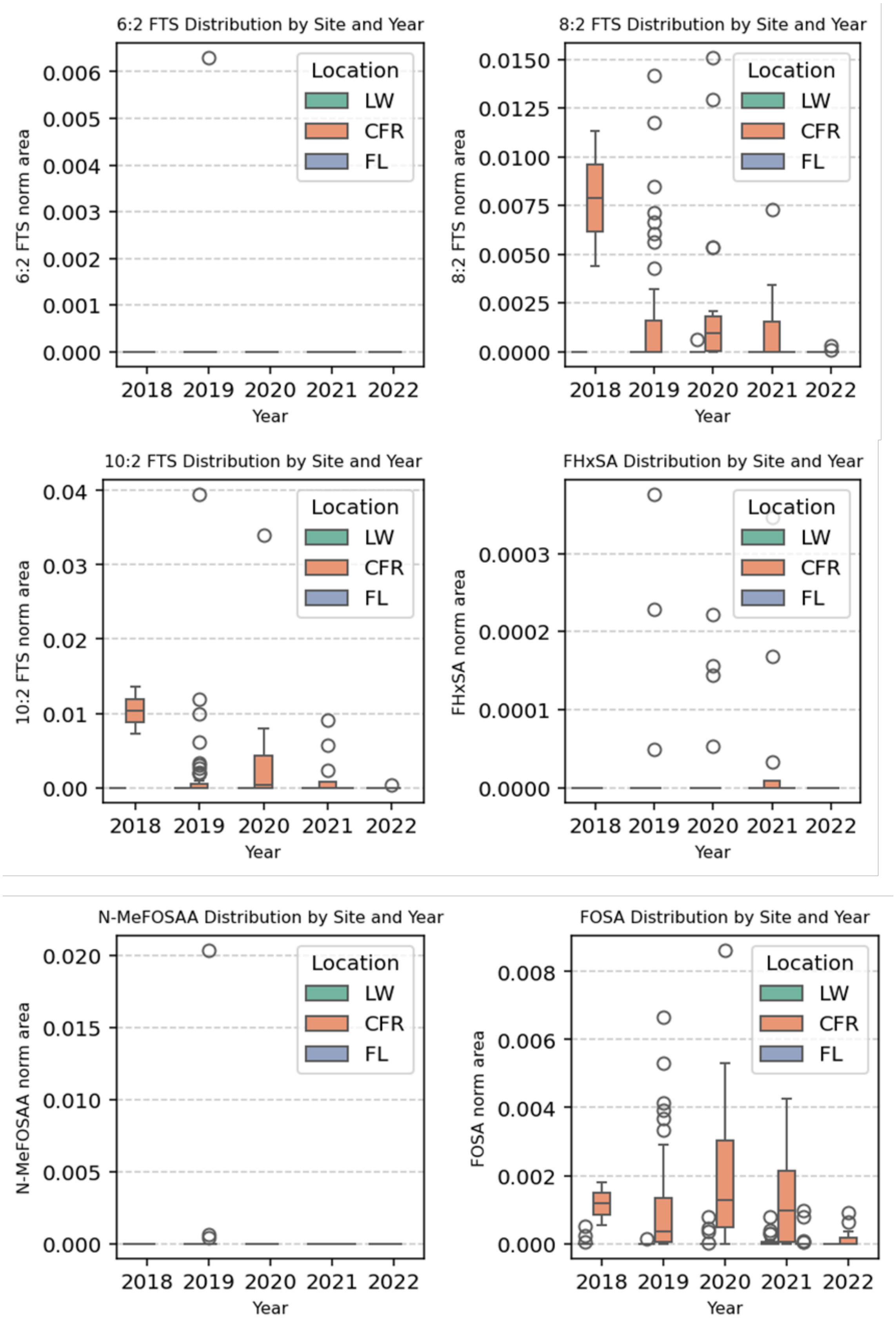
6:2 FTS Distribution by Site and Year
6:2 FTS norm area
0.000
0.001
0.002
0.003
0.004
0.005
0.006
Year
2018 2019 2020 2021 2022
Location
LW
CFR
FL
8:2 FTS Distribution by Site and Year
8:2 FTS norm area
0.0000
0.0025
0.0050
0.0075
0.0100
0.0125
0.0150
Year
2018 2019 2020 2021 2022
Location
LW
CFR
FL
10:2 FTS Distribution by Site and Year
10:2 FTS norm area
0.00
0.01
0.02
0.03
0.04
Year
2018 2019 2020 2021 2022
Location
LW
CFR
FL
FHxSA Distribution by Site and Year
FHxSA norm area
0.0000
0.0001
0.0002
0.0003
Year
2018 2019 2020 2021 2022
Location
LW
CFR
FL
N-MeFOSAA Distribution by Site and Year
N-MeFOSAA norm area
0.000
0.005
0.010
0.015
0.020
Year
2018 2019 2020 2021 2022
Location
LW
CFR
FL
FOSA Distribution by Site and Year
FOSA norm area
0.000
0.002
0.004
0.006
0.008
Year
2018 2019 2020 2021 2022
Location
LW
CFR
FL

Figure S26. Relative abundance trends for FTSs and PFASAs.

Box plots showing median and range of relative abundance for all detected fluorotelomer sulfonates (FTS) and perfluoroalkyl sulfonamides (FASAs) by watershed and year. Relative abundance is defined as the normalized peak area (total peak area divided by area of a surrogate standard)

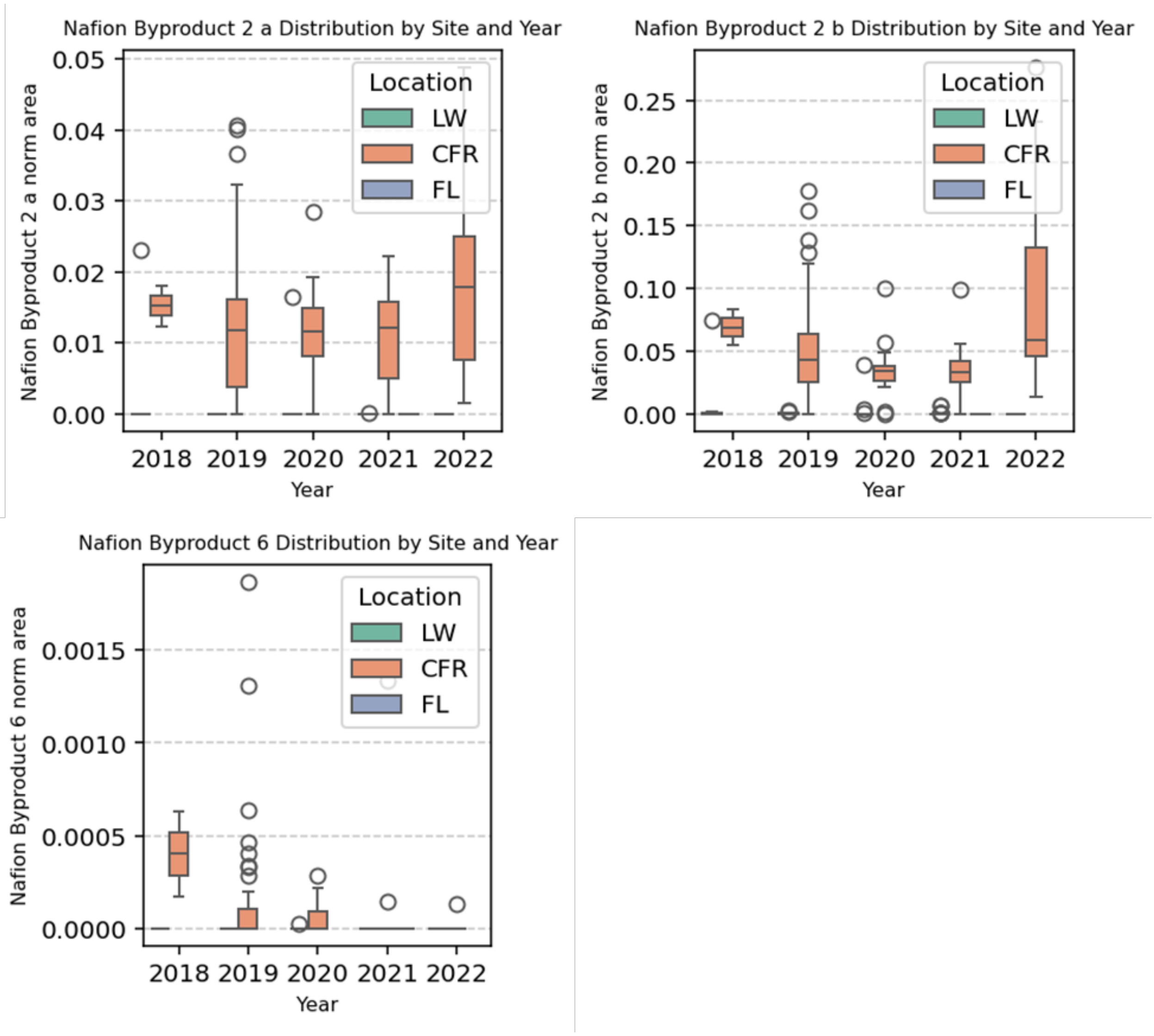

Figure S27. Relative abundance trends for Nafion byproducts.

Box plots showing median and range of relative abundance for the two previously known Nafion 2 byproducts and Nafion Byproduct 6 by watershed and year. Relative abundance is defined as the normalized peak area (total peak area divided by area of a surrogate standard)

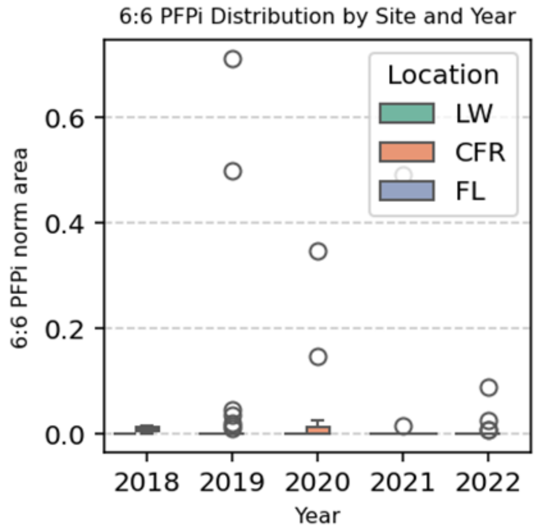
6:6 PFPi Distribution by Site and Year
6:6 PFPi norm area
0.0
0.2
0.4
0.6
2018
2019
2020
2021
2022
Year
Location
LW
CFR
FL

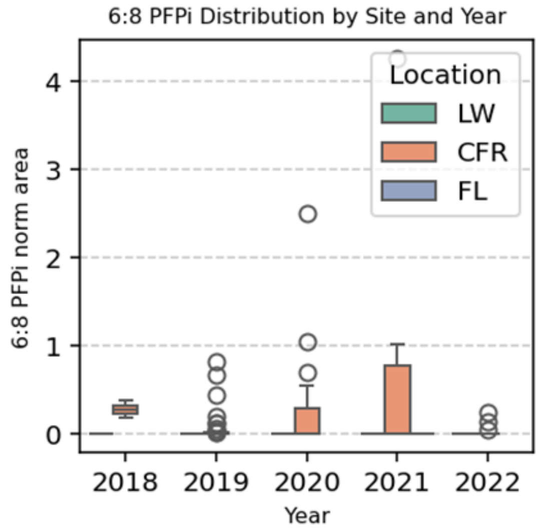
6:8 PFPi Distribution by Site and Year
6:8 PFPi norm area
0
1
2
3
4
2018
2019
2020
2021
2022
Year
Location
LW
CFR
FL

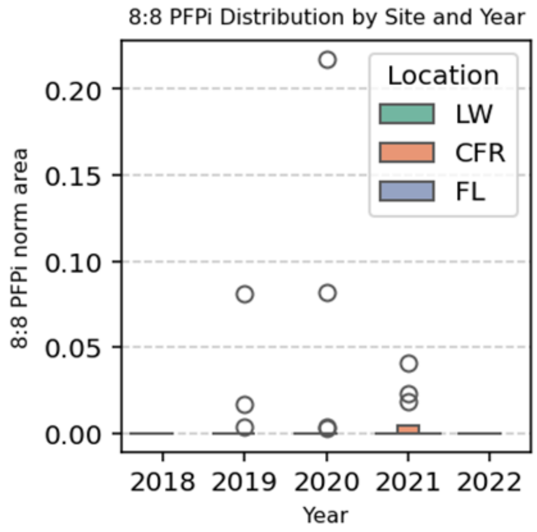
8:8 PFPi Distribution by Site and Year
8:8 PFPi norm area
0.00
0.05
0.10
0.15
0.20
2018
2019
2020
2021
2022
Year
Location
LW
CFR
FL

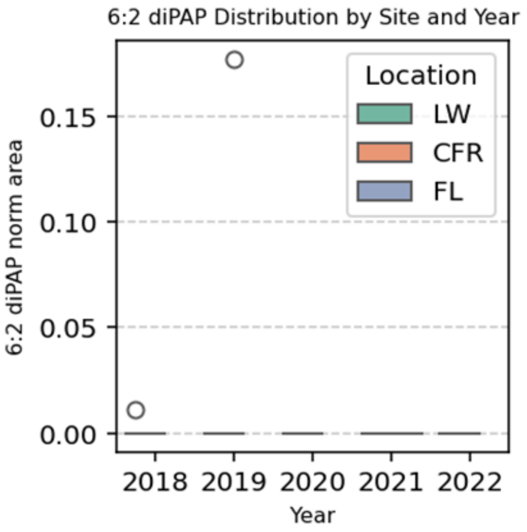
6:2 diPAP Distribution by Site and Year
6:2 diPAP norm area
0.00
0.05
0.10
0.15
2018
2019
2020
2021
2022
Year
Location
LW
CFR
FL

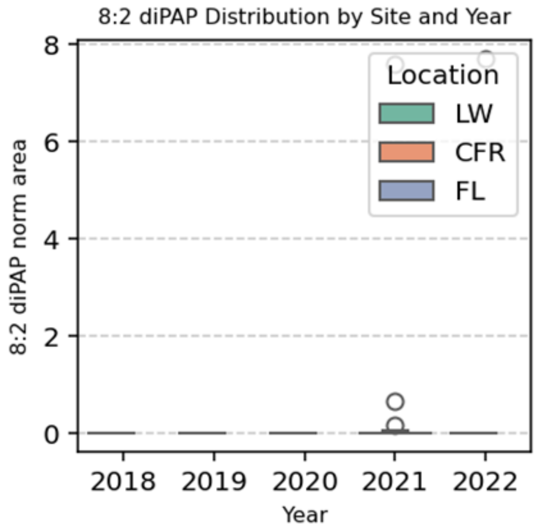
8:2 diPAP Distribution by Site and Year
8:2 diPAP norm area
0
2
4
6
8
2018
2019
2020
2021
2022
Year
Location
LW
CFR
FL

Figure S28. Relative abundance trends for PFPis and diPAPs.

Box plots showing median and range of relative abundance for all detected perfluorophosphinic acids (PFPi) and polyfluoroalkyl phosphoric acid diesters (diPAPs) by watershed and year. Relative abundance is defined as the normalized peak area (total peak area divided by area of a surrogate standard)

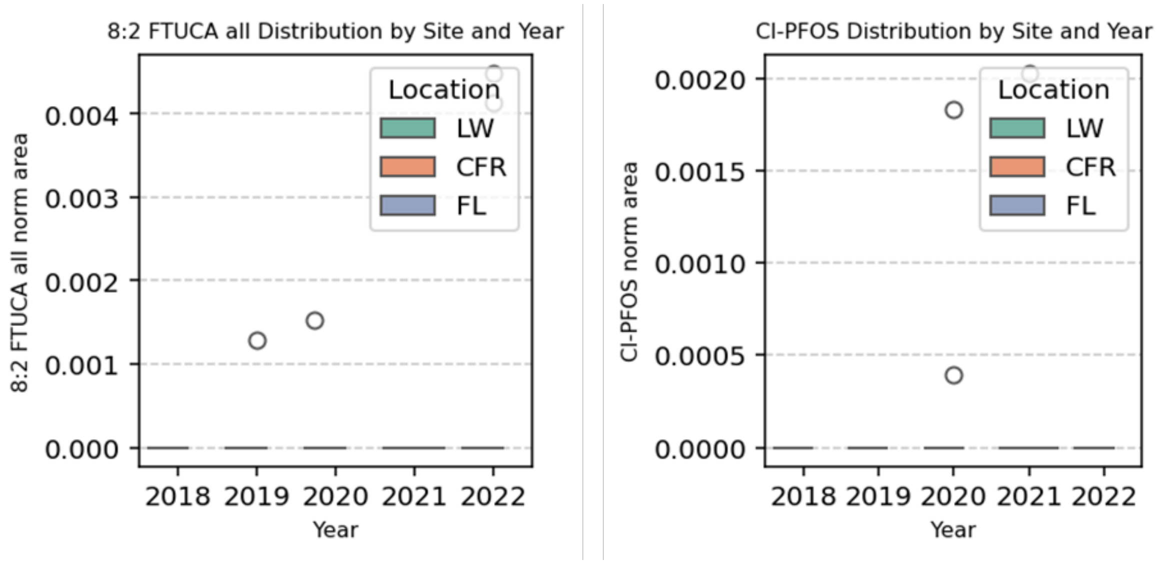

Figure S29. Relative abundance trends for 8:2 FTUCA and Cl-PFOS.

Box plots showing median and range of relative abundance for 8:2 FTUCA and Cl-PFOS by watershed and year. Relative abundance is defined as the normalized peak area (total peak area divided by area of a surrogate standard)

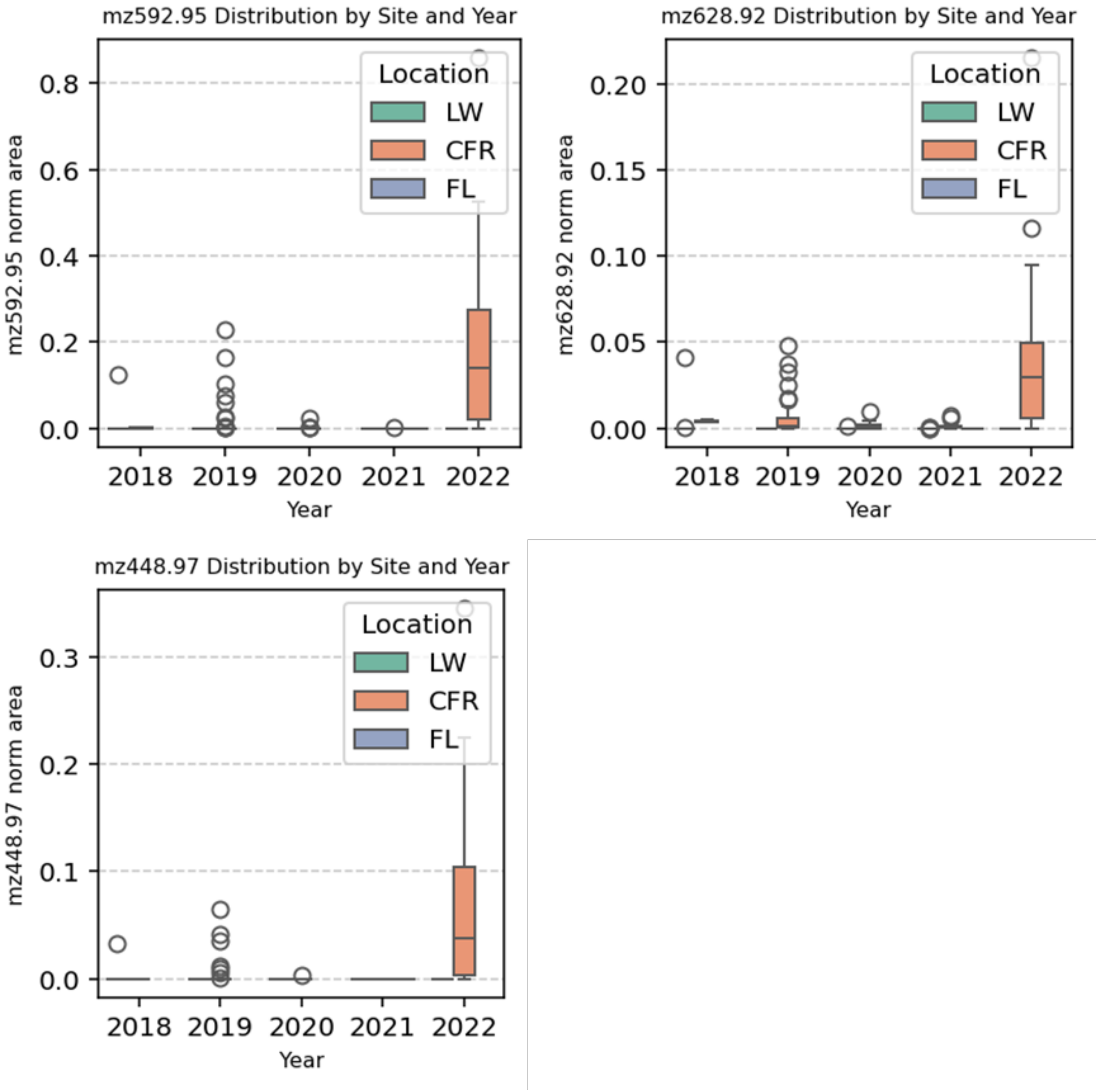

Figure S30. Relative abundance trends for novel triethers.

Box plots showing median and range of relative abundance for the novel triether compounds by watershed and year. Relative abundance is defined as the normalized peak area (total peak area divided by area of a surrogate standard)

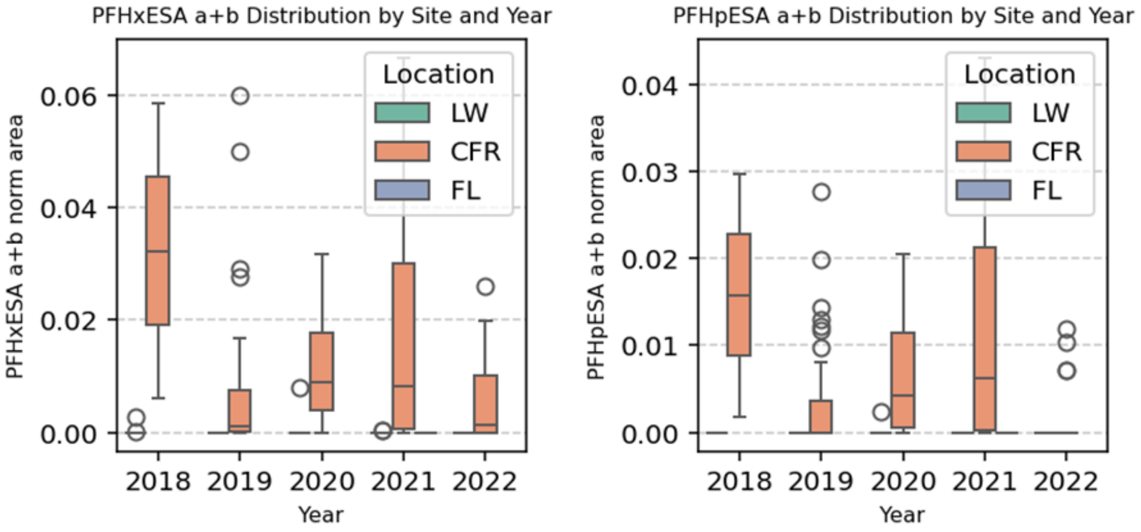

Figure S31. Relative abundance trends for novel PFAESAs.

Box plots showing median and range of relative abundance for PFHxESA and PFHpESA by watershed and year. Peak areas of all detected isomers were summed. Relative abundance is defined as the normalized peak area (total peak area divided by area of a surrogate standard)

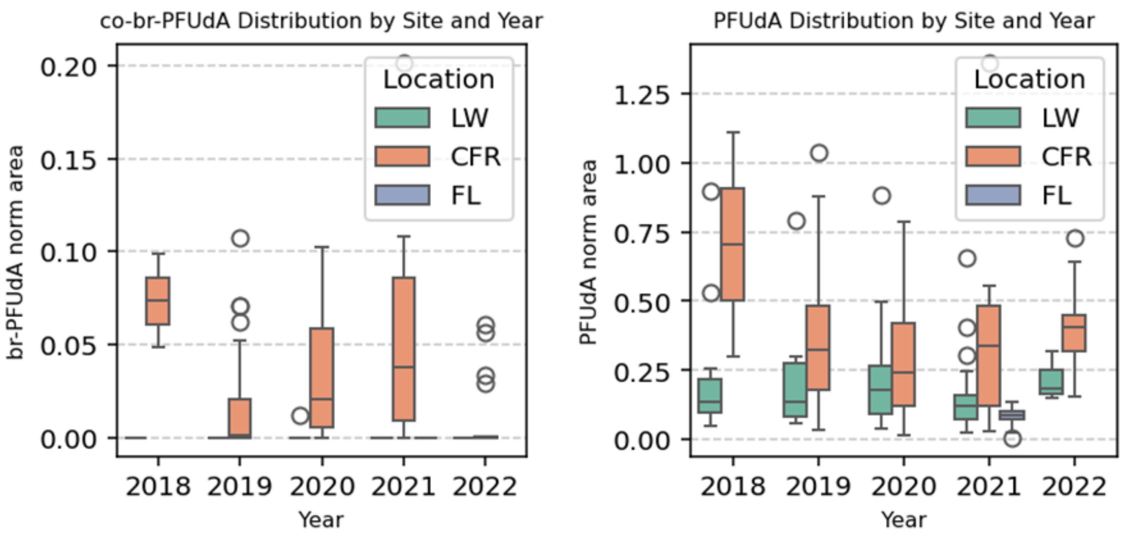

Figure S32. Relative abundance trends for PFUdA isomers.

Box plots showing median and range of relative abundance for co-br-PFUdA and linear PFUdA by watershed and year. Relative abundance is defined as the normalized peak area (total peak area divided by area of a surrogate standard)

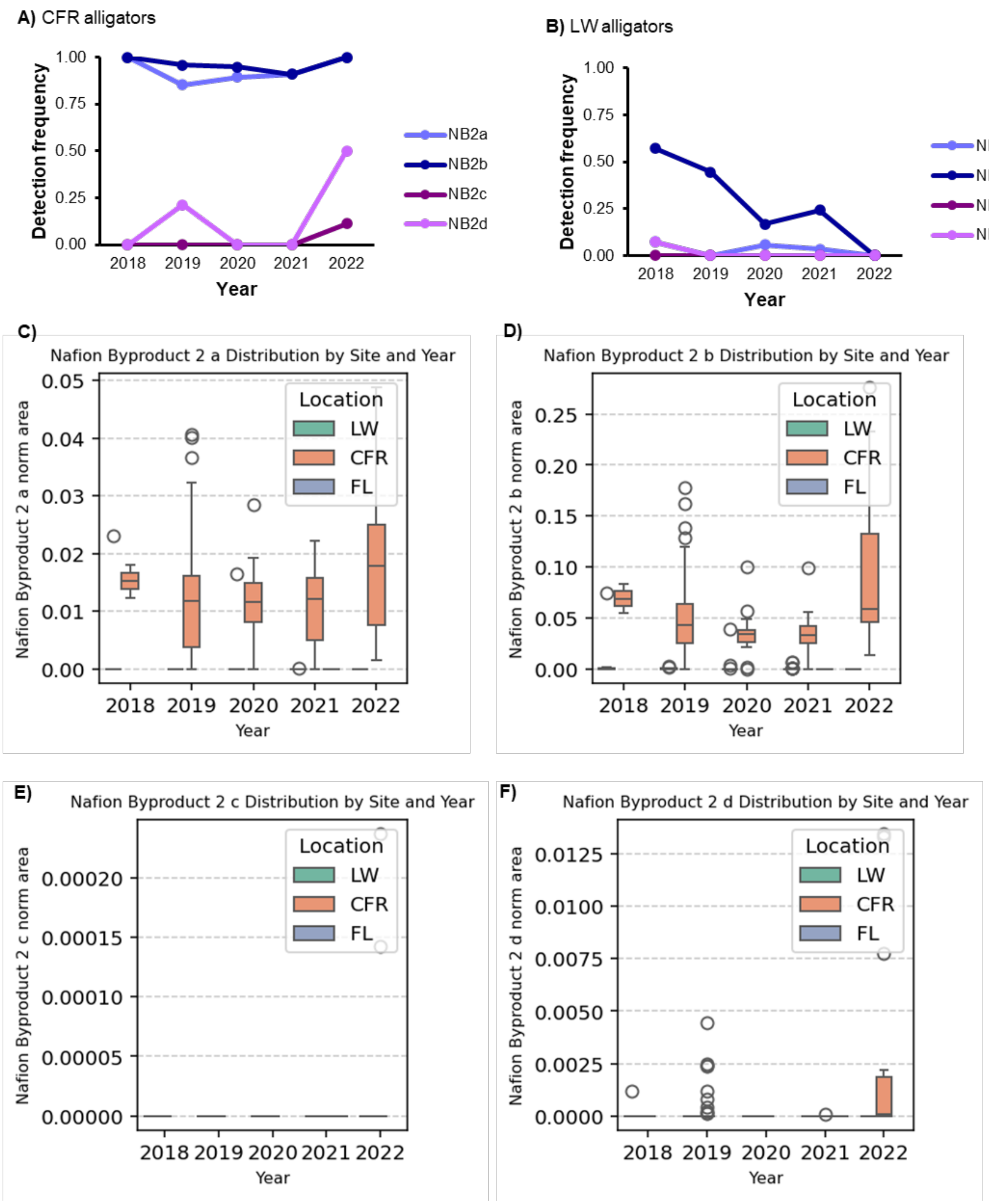

Figure S33. Relative abundance trends for PolyFESA isomers.

Unknown PolyFESA C was originally annotated as “NB2 C” and PolyFESA D was originally annotated as “NB2 D”. **A)** Detection frequency in CFR alligators **B)** Detection frequency in LW alligators **C-F)** Box plots showing median and range of relative abundance for all detected NB2 isomers by watershed and year. Relative abundance is defined as the normalized peak area (total peak area divided by area of a surrogate standard)

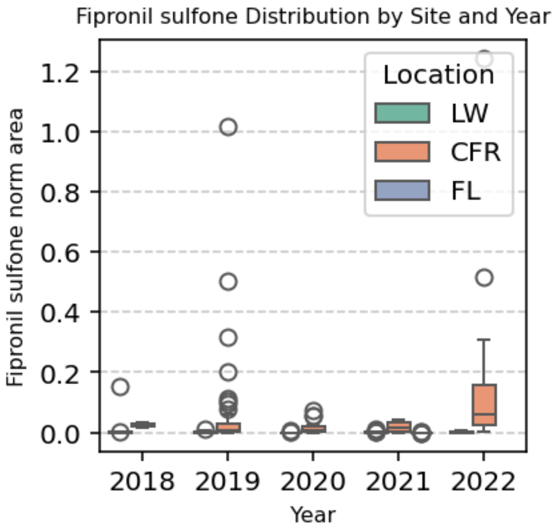

Figure S34. Relative abundance trends for fipronil sulfone.

Box plots showing median and range of relative abundance for fipronil sulfone by watershed and year. Relative abundance is defined as the normalized peak area (total peak area divided by area of a surrogate standard)

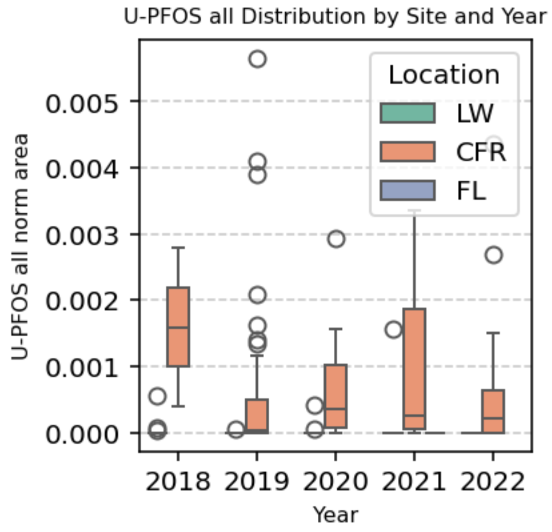

Figure S35. Relative abundance trends for U-PFOS.

Box plots showing median and range of relative abundance for U-PFOS by watershed and year. Relative abundance is defined as the normalized peak area (total peak area divided by area of a surrogate standard)

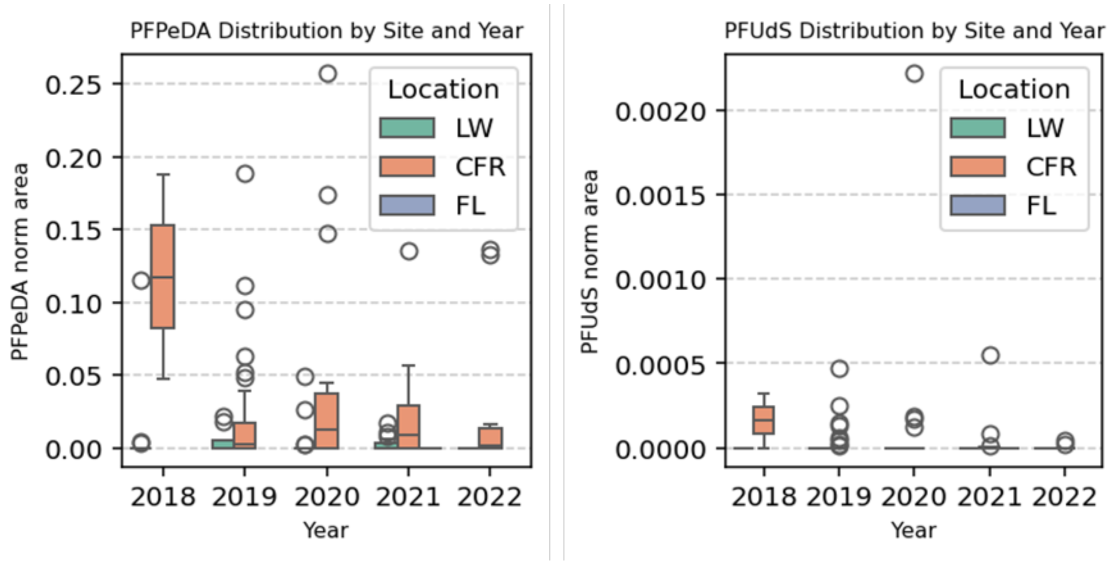

Figure S36. Relative abundance trends for PFPeDA and PFUdS.

Box plots showing median and range of relative abundance for PFPeDA and PFUdS by watershed and year. Relative abundance is defined as the normalized peak area (total peak area divided by area of a surrogate standard)

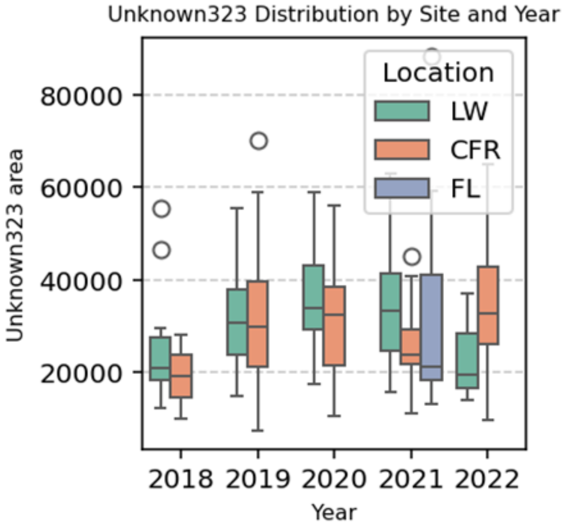
Unknown323 Distribution by Site and Year
Unknown323 area
80000
60000
40000
20000
Location
LW
CFR
FL
2018 2019 2020 2021 2022
Year

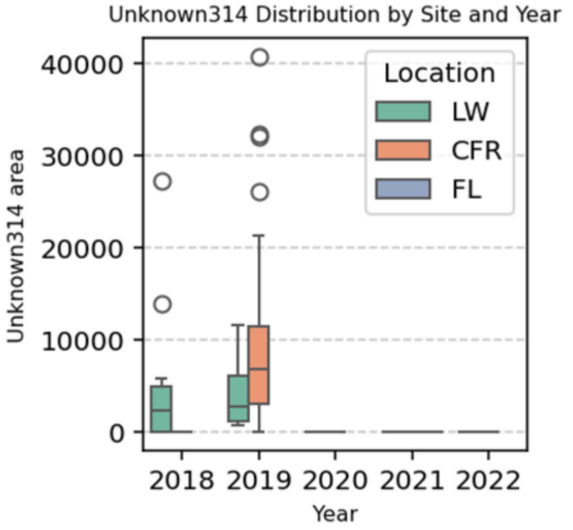
Unknown314 Distribution by Site and Year
Unknown314 area
40000
30000
20000
10000
0
Location
LW
CFR
FL
2018 2019 2020 2021 2022
Year

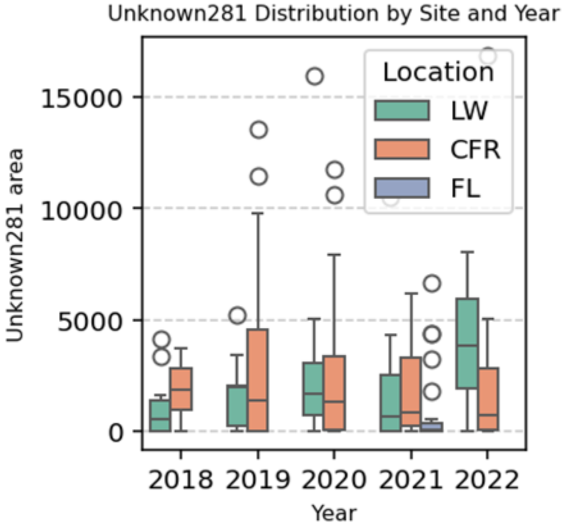
Unknown281 Distribution by Site and Year
Unknown281 area
15000
10000
5000
0
Location
LW
CFR
FL
2018 2019 2020 2021 2022
Year

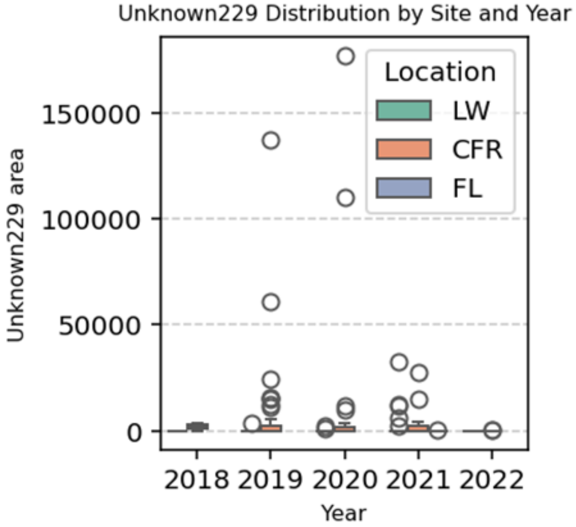
Unknown229 Distribution by Site and Year
Unknown229 area
150000
100000
50000
0
Location
LW
CFR
FL
2018 2019 2020 2021 2022
Year

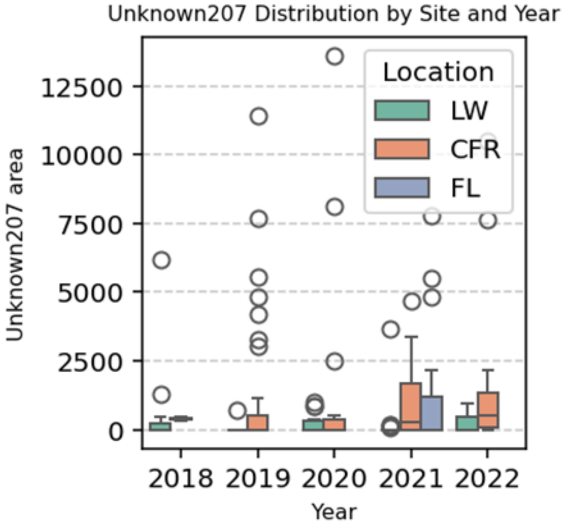
Unknown207 Distribution by Site and Year
Unknown207 area
12500
10000
7500
5000
2500
0
Location
LW
CFR
FL
2018 2019 2020 2021 2022
Year

Figure S37. Peak area trends for the unidentified features of interest.

Box plots showing median and range of peak areas for the unidentified features of interest by watershed and year.

LC-IMS-CID-HRMS Data Collection

All samples were first analyzed in MS1-only mode (no collision energy). The pooled samples and several representative individual alligator samples were reinjected using a size-dependent all-ions fragmentation method. The LC method was a 0.4 mL/min gradient with water as mobile phase A and 95% methanol and 5% water as mobile phase B, both buffered with 5 mM ammonium acetate, and increased from 10 to 100% B over 16.5 minutes (**Table SM1**). The ion source was an Agilent JetStream ESI operated in negative ion mode (**Table SM2**). The IMS drift tube used a constant voltage drop and a 60 ms maximum drift time (**Table SM3**) The QTOF was operated in *m/z* 50-1700 mode and 4-bit multiplexing was used for the MS1-only injections (**Table SM3**). For the size-dependent AIF injections, a drift time-based collision energy ramp from 2 to 60 V (**Table SM4**) was applied to alternating frames so that the larger molecules with higher drift times received a higher voltage.

Table SM1. LC gradient settings

Time (min)	% B
0	10
0.5	10
2	30
14	95
16.5 (stop time)	100
6 (post time)	10

Table SM2. Electrospray ionization source settings

Parameter	Value	Units
Gas temperature	230	°C
Drying gas flow	11	L/min
Nebulizer gas pressure	45	psig
Sheath gas temperature	350	°C
Sheath gas flow	11	L/min
Vcap	3500	V
Nozzle voltage	500	V

Table SM3. IMS-MS settings

Parameter	Value	Units
Mass range	50-1700	*m/z*
Trap fill time	3900	µs
Trap release time	300	µs
Frame rate	1	frames/sec
IM transient rate	17	transients/frame
Max drift time	60	ms
TOF transient rate	496	transients/IM transient
Multiplexing pulse sequence length	4	bit
Drift tube entrance voltage	-1574	V
Drift tube exit voltage	-224	V
Rear funnel entrance voltage	-217.5	V
Rear funnel exit voltage	-45	V

Table SM4. Collision energy ramp settings

Drift time (ms)	Collision Energy (V)
0	2
10	5
15	19
21	32
25	41
35	53
45	60
60	60

Table SM5. Mass Profiler settings

Parameter	Value
Ion intensity	>= 0.0 counts
Isotope model	Common organic molecules
Charge state	z = 1
RT tolerance	± 0.0% + 1.00 min
DT tolerance	± 1.5%
Mass tolerance	± 10.0 ppm + 2.0 mDa
Sample occurrence: Frequency	>= 10.0% across all samples
Group difference: Expression	Fold change >= 1.0

Table SM6. Fluoromatch-IM settings

Parameter	Value
Mass window	0.01 Da
CCS window	2%
Mass defect flag: upper	0.12
Mass defect flag: lower	-0.11
Polarity	Neg
